# Interpreting life-history traits, seasonal cycles, and coastal climate from an intertidal mussel species: Insights from 9000 years of synthesized stable isotope data

**DOI:** 10.1371/journal.pone.0302945

**Published:** 2024-05-22

**Authors:** Veronica Padilla Vriesman, Jessica R. Bean, Hannah M. Palmer, Roxanne M. W. Banker

**Affiliations:** 1 Department of Geosciences, Oberlin College, Oberlin, Ohio, United States of America; 2 Department of Earth and Planetary Sciences, University of California, Davis, Davis, California, United States of America; 3 University of California Museum of Paleontology, University of California, Berkeley, Berkeley, California, United States of America; 4 Department of Geoscience, University of Nevada Las Vegas, Las Vegas, Nevada, United States of America; Uppsala Universitet, SWEDEN

## Abstract

Understanding past coastal variability is valuable for contextualizing modern changes in coastal settings, yet existing Holocene paleoceanographic records for the North American Pacific Coast commonly originate from offshore marine sediments and may not represent the dynamic coastal environment. A potential archive of eastern Pacific Coast environmental variability is the intertidal mussel species *Mytilus californianus*. Archaeologists have collected copious stable isotopic (δ^18^O and δ^13^C) data from *M*. *californianus* shells to study human history at California’s Channel Islands. When analyzed together, these isotopic data provide windows into 9000 years of Holocene isotopic variability and *M*. *californianus* life history. Here we synthesize over 6000 δ^18^O and δ^13^C data points from 13 published studies to investigate *M*. *californianus* shell isotopic variability across ontogenetic, geographic, seasonal, and millennial scales. Our analyses show that *M*. *californianus* may grow and record environmental information more irregularly than expected due to the competing influences of calcification, ontogeny, metabolism, and habitat. Stable isotope profiles with five or more subsamples per shell recorded environmental information ranging from seasonal to millennial scales, depending on the number of shells analyzed and the resolution of isotopic subsampling. Individual shell profiles contained seasonal cycles and an accurate inferred annual temperature range of ~ 5°C, although ontogenetic growth reduction obscured seasonal signals as organisms aged. Collectively, the mussel shell record reflected millennial-scale climate variability and an overall 0.52‰ depletion in δ^18^O_shell_ from 8800 BP to the present. The archive also revealed local-scale oceanographic variability in the form of a warmer coastal mainland δ^18^O_shell_ signal (-0.32‰) compared to a cooler offshore islands δ^18^O_shell_ signal (0.33‰). While *M*. *californianus* is a promising coastal archive, we emphasize the need for high-resolution subsampling from multiple individuals to disentangle impacts of calcification, metabolism, ontogeny, and habitat and more accurately infer environmental and biological patterns recorded by an intertidal species.

## Introduction

Anthropogenic changes in sea surface temperature (SST), ocean chemistry, and carbon cycling have been documented in nearshore surface waters globally [1–3]. Changing environmental conditions are particularly threatening to complex coastal ecosystems, which are highly sensitive to sea-level rise, storms, nutrient runoff, marine heat waves, and ecological regime shifts [4–7]. Concern about the vulnerability of nearshore ecosystems to environmental perturbation is amplified by the cultural and economic importance of coastlines, as they are densely populated regions that have been influenced by human-environment interactions for thousands of years [8, 9]. The social and economic relevance of coastal zones continues to expand; nearly half of the global population is expected to live within 100 km of a coastline within the next decade [6, 10, 11]. As such, coastal archives of past climate and ecological change are of particular importance for understanding past natural baselines and estimating the impacts of humans within coastal systems [8, 12–14]. Biogenic coastal archives can reveal valuable information about broader scale climate oscillations [15–18] to supplement the vast collections of offshore archives, such as deep-sea marine sediments and corals, and terrestrial archives, such as speleothems, lake records, and ice cores [19–24].

Here, we investigate the potential of *Mytilus californianus* (California mussel) shells as a high-resolution coastal archive of Holocene environmental variability for the Pacific Coast of North America. *Mytilus californianus* shell geochemistry could be a promising source of regional coastal climate proxies for California, USA due to its abundance and prevalence in the archaeological record [25], but geochemical data from *M*. *californianus* have not been comprehensively analyzed across multiple time intervals and regions; prior studies have focused on a single locality, or set of localities, and/or a single time interval. We employ a multi-scaled analysis of previously published stable isotope datasets of Holocene-aged *M*. *californianus* shells from southern California to assess the utility of *M*. *californianus* as an accurate recorder of seasonality and climate variability, and to document regional climate variability across both spatial (e.g., islands versus mainland) and temporal scales (e.g., seasonal versus millennial). By analyzing ontogenetic, geographic, seasonal, and millennial scales of stable isotope variability, we contribute a newly synthesized 9000-year-long, snapshot-based spatio-temporal record of *M*. *californianus* with novel insights for shell sampling and analysis. We integrate and analyze these data in a format that is now useful for inferring paleoceanographic conditions and clarifying mussel life-history traits at various spatio-temporal scales. The synthesis of this assembled archive also reduces the need for further invasive sampling from culturally significant midden sites [26, 27].

### Ecology, distribution, and life history of *Mytilus californianus*

*Mytilus californianus* is dominant in modern rocky intertidal ecosystems spanning 25° of latitude along the northeastern Pacific Coast from the Aleutian Islands of Alaska, USA to Baja California, Mexico [28]. As a foundation species, *M*. *californianus* forms a habitat for hundreds of marine taxa, provides a food source for coastal predators, and filters particulate matter from the intertidal water column [28–30]. *Mytilus californianus* beds are extensive and dominant in exposed, open-ocean environments with heavy wave action due to its strong, tri-layered shell and thick byssal threads [31, 32]. Because of its widespread biogeographic distribution and its role in intertidal food webs, *M*. *californianus* has been a foundational study organism in the fields of intertidal ecology and marine invertebrate physiology since the mid-20th century [28, 31, 33–41]. Despite the attention *M*. *californianus* has received, its life history remains enigmatic; its lifespan is uncertain, although it is thought to range from 10 to 100 years depending on environmental conditions and disturbance levels [31, 42]. Shell growth rates are variable across both micro-scale (e.g., tidal) and macro-scale (e.g., latitudinal) gradients that control conditions such as water temperature, wave action, and immersion time [29, 40, 43]. Estimating shell growth rates and longevity is further complicated by the lack of time-calibrated periodic growth bands [44]. Despite these complexities, previous studies have documented growth patterns of *M*. *californianus* in southern California populations; Ford et al. (2010) found that *M*. *californianus* shell length grew continuously over the course of a 382-day outplant experiment in San Diego, which is advantageous for reconstructing minimum and maximum temperatures over the course of a year from shell isotopic records [42]. Additionally, Ferguson et al. (2013) found evidence for continuous calcification without growth shutdown in *M*. *californianus* throughout the year at sites in southern California, USA and Baja California, Mexico [45]. There is also general agreement in the mytilid literature that adult *M*. *californianus* shells accrete approximately 2–5 mm at their terminal growth margin per month in southern Californian environments [25, 31, 34], where they grow more rapidly in seawater temperatures ranging from 15° to 19°C [29]. Continuous annual growth patterns and the relationship between temperature and growth rate are specific to southern California since the upwelling and temperature regimes differ dramatically for regions north of Point Conception, resulting in significantly slower growth rates for marine calcifying biota in the central and northern portions of the California Current System [29, 40, 44, 46].

### *Mytilus californianus* and human history

In addition to its significant role in structuring the intertidal ecosystem, *M*. *californianus* has been a culturally important species and major food source for Indigenous peoples throughout the Holocene. *Mytilus californianus* shells are found in northeastern Pacific Coast shell middens at a higher density than any other bivalve species [25]. This species is particularly abundant in the shell middens of the Channel Islands, California, USA due to the populous Chumash civilizations that inhabited southern California throughout the Holocene [25, 47, 48]. Prior to European colonization, the Chumash peoples of present-day southern California had established 22 major villages on Santa Cruz, San Miguel, and Santa Rosa Islands, built extensive trade networks throughout coastal California and all nine Channel Islands, and reached the largest populations among hunter-gatherer civilizations known to published records [49]. The dominant presence of *M*. *californianus* shells in Channel Islands shell middens across the Holocene indicates that the Chumash groups heavily and continuously harvested *M*. *californianus* as a significant food source [48, 50].

Exceptional preservation of Channel Islands shell middens has attracted the attention of archaeologists and anthropologists for the past two centuries, producing an extensive collection of *M*. *californianus* shells extracted from the middens. Archaeologists commonly apply stable isotopic analysis to midden shells to reconstruct the season of harvest, which is useful for investigating questions about human migration patterns, site usage, and cultural traditions [51–55]. When analyzed collectively, the ample stable isotope records from archaeological *M*. *californianus* shells have the potential to provide an archive of coastal climate variability, seasonality, and shell life-history traits through time. In general, the vast isotopic data from previously excavated collections of archaeological shells have the potential to be valuable paleo-archives. Here, we test the use of *M*. *californianus* data from the Channel Islands middens to investigate the utility of this species as a record of environmental conditions across the Holocene.

### Shell chemistry of *Mytilus californianus*

Species-specific relationships between δ^18^O_shell_ and sea surface temperature (SST) and potential covariation in δ^13^C_shell_ and the δ^13^C of dissolved inorganic carbon (DIC) of seawater allow for paleoenvironmental reconstruction from *M*. *californianus* shell carbonate. When sampled serially along the ontogenetic growth trajectory (from umbo to commissure), δ^18^O_shell_ has been found to be a reliable record of mean annual temperature and mean annual temperature ranges in modern *M*. *californianus* shells from San Diego, with a slight and consistent temperature-dependent enrichment in ^18^O_shell_ by 0.2 to 0.5‰ relative to predicted δ^18^O_equilibrium_ [42]. Interpretation of δ^13^C_shell_ is more complex and inconsistent due to the incorporation of metabolic (respired) carbon in the shell mineral. In molluscs, δ^13^C_shell_ typically decreases with ontogeny as physiology changes with age [56]. For *Mytilus* in particular, up to 10% of isotopically light respired carbon can be incorporated into the shell during calcification, and metabolic rate can vary throughout ontogeny [45, 57], hindering the use of δ^13^C_shell_ profiles as direct proxies of the environment. However, significant changes in δ^13^C_shell_ through time can also indicate broad shifts in ocean carbonate chemistry, as documented by a negative trend in modern δ^13^C_shell_ in *M*. *californianus* relative to δ^13^C_shell_ of archival and archaeological *M*. *californianus* shells collected in Washington State [58]. In addition to assessing long-term changes in ocean carbonate chemistry, δ^13^C_shell_ profiles from multiple individuals from the same locality could be used to track short-term, upwelling-driven changes in ocean chemistry on a seasonal scale since upwelled water delivers nutrients and respired, isotopically light carbon to surface waters [45, 59], although the relationship between δ^13^C_shell_ and δ^13^C of DIC in seawater is inconsistent [45, 60, 61]. While Killingley and Berger (1979) found remarkable correlation and covariation between upwelling indices and δ^13^C_shell_ for San Diego *M*. *californianus* shells, more recent work did not corroborate these patterns in modern *M*. *californianus* shells from the same locality [45, 59].

Diagenesis is a potential concern when interpreting stable isotope and trace element data from archaeological or fossilized carbonates. All non-modern *M*. *californianus* shells analyzed in this study are from shell middens, which structurally and chemically favor preservation since biogenic shells in a midden deposit serve as a natural, carbonate-rich buffer to dissolution and the layers of a midden act as a barrier against erosion [62]. Additionally, *M*. *californianus* has an outer layer of calcite [44, 63], which is the more stable form of calcium carbonate and therefore less susceptible to alteration. All sub-samples analyzed here are from the outer calcite layer of *M*. *californianu*s shells, and all midden shell data were sourced from studies that methodically addressed diagenesis by performing X-ray diffraction analysis and/or HCl etching to avoid sampling diagenetically altered shell carbonate prior to stable isotope analysis [64, 65].

## Methods

### Study area

The California Current System (CCS) influences patterns of sea surface temperature (SST), upwelling intensity, and nutrient concentrations in southern California. The CCS comprises the cold California Current, the warm north-flowing California Undercurrent (subsurface), the warm north-flowing Davidson Current (surface), and the Southern California Eddy [46, 66–68]. The dominant California Current flows southward along the coastline of northern California parallel to the alongshore winds, resulting in strong Ekman transport and intense upwelling near the coast of northern and central California [46, 67]. South of Point Conception, the coastline morphology changes such that the current no longer runs parallel to alongshore winds, which results in weaker Ekman transport and upwelling along southern California. The mild upwelling produces a warmer SST regime along the southern California coast relative to the northern CCS. Wind forcing and upwelling is strongest during the summer in all portions of the CCS [46], but seasonal variability is slightly less pronounced south of Point Conception, resulting in a warm zone and a weaker yet more stable upwelling regime off the coast of southern California.

**Fig 1:**
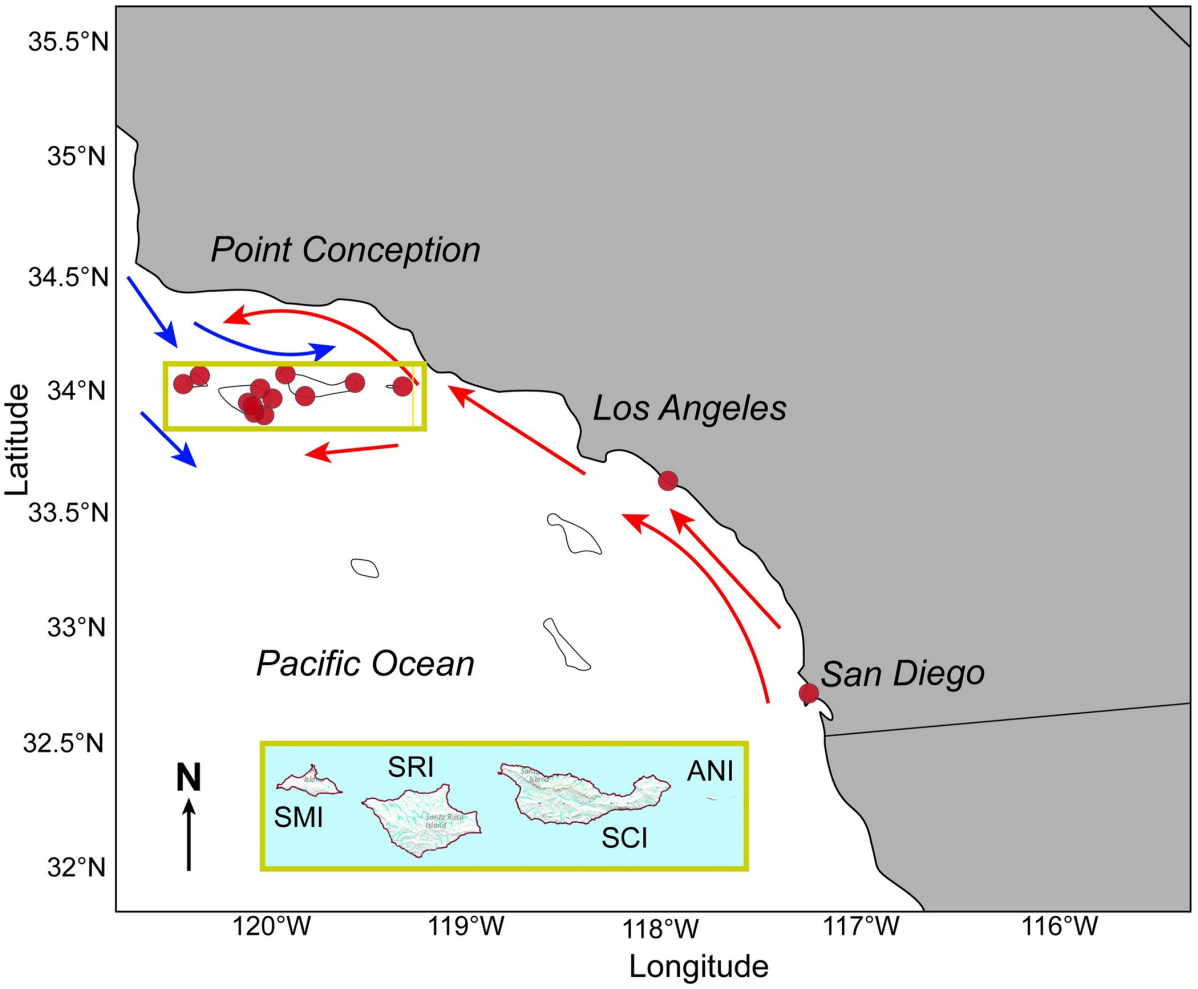
Map of study region in southern California for illustrative purposes only. Collection sites (red dots) are indicated at northern Channel Islands (San Miguel Island, Santa Rosa Island, Santa Cruz Island, and Anacapa Island), Newport Beach, and San Diego. Inset map of northern Channel Islands modified from USGS National Map Viewer.

This study uses only *M*. *californianus* shells collected in the southern portion of the CCS, south of Point Conception and north of Baja California (Fig 1), due to the abundance of *M*. *californianus* records from this region. Specific collection sites are located on the northern Channel Islands (San Miguel Island, Santa Rosa Island, Santa Cruz Island, and Anacapa Island), Newport Beach, and San Diego. Channel Islands mussels were more likely to experience relatively lower temperatures from the cold California Current, while mussels from Newport Beach and San Diego may have experienced warmer water on the order of ~ 3°C delivered by the California Undercurrent and Davidson Current [69]. The temperature differential between the islands and mainland sites is compounded by considerable small-scale oceanographic variability among the northern Channel Islands due to ocean current activity, which produces a warm-cool SST gradient from east to west [69–72]. At Santa Cruz Island, for example, mean SSTs have been lower on the west coast than the southeast coast throughout the Holocene [72]. Mean SSTs at San Miguel Island are ~ 1 to 4°C lower than those at Anacapa Island [73]. The dynamic nature of the intertidal zone is another source of micro-scale temperature variability across tidal heights even within a single locality. In addition to multi-scaled spatial variability, seasonal patterns result in a ~ 5°C annual temperature range [54, 74]. For example, at Anacapa Island, SST alternates between warming (~ 16°C to 19°C) from late June through early September and cooling (~ 19°C to 15°C) from late September to early March [53]. Seasonal cycles are similar throughout the northern Channel Islands (i.e., cooling and warming periods occur relatively synchronously for all four islands) due to the seasonally driven upwelling regime [73]. No permits were required for our study since no field sites were accessed; all data were sourced from previously published studies.

We analyzed Channel Islands shells separately from mainland California shells to determine whether the Channel Islands produced higher mean δ^18^O_shell_ values as expected due to cooler seawater conditions farther offshore. Comparing mainland and island data also provides the opportunity to examine potential δ^18^O_shell_ and δ^13^C_shell_ shifts related to coastal versus offshore differences in salinity due to precipitation and freshwater runoff.

### *Mytilus californianus* shell records

We used Web of Science to search for relevant literature in geochemical, archaeological, paleontological, paleoceanographic, and sclerochronological journals using combinations of the following keywords: California mussel, *Mytilus californianus*, stable isotope data, oxygen isotopes (δ^18^O), and carbon isotopes (δ^13^C). We included oxygen and carbon isotope data from *M*. *californianus* shells that met the following criteria for integration and further analysis:

- all shells were definitively identified by the original authors as *M*. *californianus*.
- all shells were sourced from southern California (south of Point Conception), including the Channel Islands, either collected live from the intertidal zone or excavated from a coastal shell midden or outcrop.
- all mussels lived during the Holocene (geologically younger than 11,700 years old).
- all shells have multiple (two or more) stable isotope subsamples per shell along the ontogenetic growth trajectory with a known sampling direction (i.e., stable isotope data is presented in relation to the axis of shell growth to infer the directionality of stable isotope trends in individual shell profiles).
- non-modern shells were examined with X-ray diffraction and/or treated with HCl etching prior to stable isotope analysis to avoid sampling diagenetically altered shell material.
- all shells were complete or nearly complete valves (no fragments or shells made into tools or jewelry).
- non-modern shells were published with geographic locations and associated radiocarbon ages from a dated section of a midden to confidently place specimens in temporal context, ± 30 years.
- modern shells had a known collection date (month/day/year) and latitude/longitude.
  While we intended to include fossil specimens in this study, we found no published records of stable isotope data from fossilized *M*. *californianus* shells from this region and time interval (but see Dodd (1966) for stable isotopic analysis of Pleistocene *Mytilus* shells from other regions of California [75]). We included no summary statistics in our dataset; we only entered shells that reported the full suite of δ^18^O_shell_ and δ^13^C_shell_ sub-sampled values. The resultant assembled dataset contains 6036 oxygen and carbon stable isotope values from 411 *M*. *californianus* shells integrated from 13 published studies (S1 File). The majority (10 of 13 studies) utilized archaeological shells to reconstruct patterns of early human settlement or foraging behavior [51–53, 72, 76–81], while the other three studies used modern *M*. *californianus* shells to calibrate stable isotope-based environmental proxies [42, 45] or test the influence of tidal height on shell geochemistry [42, 74]. Summary information, including study objectives and findings, of all 13 papers are presented in Table 1. For each individual shell that met our criteria, we assigned it a unique shell ID number in Excel and then entered δ^18^O_shell_ and δ^13^C_shel_ values for each subsample in a unique row associated with that shell ID. In addition to stable isotope data, the synthesized dataset also contains the number of subsamples per shell, the citation of the primary publication, subsample label or distance along shell (mm), age in years before present (BP), specimen type (midden or modern), location (islands or mainland), site (specific island or city), latitude, and longitude. The subsample distance along the shell is unrelated to total shell length. In most cases, the total shell length was not provided, although we included it whenever it was reported by primary authors (S1 File). No permits were required for the described study, which complied with all relevant regulations.

**Table 1:** Summary of the 13 papers from which data are sourced for this study.

Citation	Site(s)	Age or age range	Purpose and findings
Ford et al., 2010 [42]	San Diego	Modern	Experimental geochemical study to test the effects of intertidal position and shell growth rate on shell δ^18^O to assess its utility as a temperature proxy in San Diego. Mussel shell δ^18^O was found to reliably represent mean annual temperature at this site, although shell δ^18^O was often enriched in ^18^O relative to ambient seawater.
Ferguson et al., 2013 [45]	San Diego, Newport Beach	Modern	Geochemical study to test whether carbon isotopes in mussel shells can be used as relative indicators of upwelling in southern California. Shell Δ^14^C can serve as a proxy for upwelling strength, but shell δ^13^C is less reliable.
Glassow et al., 1994 [51]	Santa Cruz Island	Middle Holocene (5900–4500 BP)	Archaeological study to investigate the increase in red abalone shells relative to black abalone shells in Santa Cruz middens during this time interval. Mussel shell δ^18^O revealed lower sea surface temperature conditions during this time, which the authors identify as the cause for the increase in red abalone shells (a cool-water taxon).
Rick et al., 2006 [52]	Santa Rosa Island	Middle Holocene (6000 BP, 4300 BP)	Archaeological study of shellfish harvesting patterns on Santa Rosa Island to investigate environmental and cultural context of changes in shellfish harvesting preferences from 6000 BP to 4300 BP. The authors attribute shifts in foraging patterns to an inferred 2°C increase in sea surface temperature inferred from mussel shell δ^18^O.
Jew and Rick, 2014 [53]	Anacapa Island	Late Holocene (3010 BP)	Archaeological study to reconstruct human foraging and site occupation patterns at Anacapa Island during the late Holocene. Mussel shell δ^18^O implied year-round shellfish harvesting, indicating that Anacapa Island was occupied more heavily and consistently than expected.
Flores, 2017 [72]	Santa Cruz Island	Late Holocene (2200–500 BP)	Archaeological study to assess the effects of oceanographic conditions on late Holocene mussel harvesting patterns at Santa Cruz Island. The author inferred consistent upwelling along the west coast of Santa Cruz Island during the late Holocene.
Jazwa et al., 2020 [74]	Santa Rosa Island	Modern	Experimental archaeological study to assess the effects of tidal height on mussel shell δ^18^O, δ^13^C, and shell length. Out of the measured factors, tidal height had the strongest effect on shell length.
Glassow et al., 2012 [76]	Santa Cruz Island	Middle Holocene (6300–5300 BP)	Archaeological study to reconstruct seawater temperatures along the western coast of Santa Cruz Island to contextualize the increase in red abalone in middens from this time interval. The temperature conditions inferred from mussel shell δ^18^O revealed cooler seawater conditions during this time, suggesting that red abalone was more accessible for harvesting in intertidal waters.
Jazwa and Kennett, 2016 [77]	San Miguel Island	Late to middle Holocene (75500–3600 BP and after 3600 BP)	Archaeological study to reconstruct the seasonality of shellfish harvesting at western San Miguel Island during the middle and late Holocene. Seasonal interpretations of shell δ^18^O indicated that mussel shell harvesting took place in the spring.
Jew et al., 2013 a [78]	San Miguel Island	Early Holocene (8800 BP)	Archaeological study to reconstruct season of harvest and human migration or sedentism across a range of sites on San Miguel Island at one time point. The authors interpreted sites with year-round shell harvesting as "residential basecamps", while other sites were likely used seasonally.
Jew et al., 2013 b [79]	San Miguel Island	Early Holocene (8200 BP)	Archaeological study to examine shell growth rates, oxygen isotope values, and season of harvest interpretations in mussel shells from 8200 BP at one site on the western edge of San Miguel Island. Interpretations ranged from short-term occupation to multi-seasonal occupation, depending on shell length and growth rate.
Robbins et al., 2013 [80]	Santa Rosa Island	Middle Holocene (7000 BP—3700 BP)	Archaeological study to investigate relationships between midden faunal assemblages and inferred seawater temperature patterns at one site on the southern coast of Santa Rosa Island. Temperature oscillations at this site coincide with shifts in faunal assemblages, suggesting that human foraging patterns were strongly controlled by environmental conditions. This study included one modern shell from San Miguel Island for comparison.
Kennett, 1998 [81]	San Miguel Island, Santa Cruz Island, Santa Rosa Island	Holocene (11,500 BP through modern)	Archaeological study to investigate the history of human settlement, foraging, and migration patterns at the northern Channel Islands prior to European colonization. Midden analysis revealed a trend towards increasing sedentism and fluctuations in hunting and foraging preferences in response to environmental and societal disturbances.

### Stable isotope sampling techniques used by primary authors

Shell profiles included in our data synthesis range from 2 to 60 subsamples per individual, representing 2 to 118 mm worth of shell, respectively. Subsamples are spaced evenly, 1 to 3 mm apart, depending on the study. The subsample spacing is reported serially in mm for each individual whenever this information was reported by primary authors.

Many archaeological studies apply the terminal growth band (TGB) sampling approach to infer the season of harvest [77–79, 82]. For individual shells with fewer sub-samples (< 5) analyzed here, the primary authors performed TGB sub-sampling nearest the commissure. To determine whether δ^18^O_shell_-inferred SST was increasing (indicative of spring-summer) or decreasing (indicative of fall-winter) during the time of collection, primary authors drilled an additional one to three sub-samples of shell material that precipitated just before the TGB, a sampling approach written as TGB + *n*, where *n* = number of additional sub-samples [78, 79]. For example, TGB + 1 indicates that one sub-sample was taken at the terminal growth band of the shell, and one additional sub-sample was drilled ~ 2–3 mm umbo-ward of the TGB, producing a two-sample shell profile used to determine the season or conditions occurring at the end of the individual’s life. This commonly used subsampling strategy is based on the estimate of ~ 2–3 mm of growth per 1–2 months in adult *M*. *californianus* shells growing in southern California [25, 29, 34, 74, 77].

### Categorization and analysis of shell records

After compiling stable isotope data from all shells that met our criteria, we categorized these data for analysis based on sample age, location, and sub-sampling strategy. For geographic categorization, data were binned according to their individual site (San Miguel Island, Santa Rosa Island, Santa Cruz Island, Anacapa Island, San Diego, Newport Beach) and whether they were from an island or the mainland. For temporal comparisons, data were binned both by millennium (e.g., within the past 1000 years, 2000, 3000, etc. up to 9000 BP) and by sub-epoch (early, mid, or late Holocene) according to the Holocene sub-epoch boundaries formally defined by Walker et al. (2019): early Holocene (11,750–8200 BP), mid Holocene (8200–4200 BP), and late Holocene (4200–0 BP) [83]. For late Holocene shell samples, we distinguished between sample types to compare late Holocene midden samples and late Holocene modern, live-collected samples (S1 File).

Individual shells were categorized for analysis based on subsampling strategy: long profiles (15–60 sub-samples per shell, or 28–118 mm of growth), medium profiles (5–14 sub-samples per shell, or 8–26 mm of growth), and short shell profiles (2–4 sub-samples per shell, or the last 4–6 mm of growth per individual shell). Sampling strategies were categorized in this way for three reasons: (1) to run analyses using the longest and highest-resolution shell records to investigate life-history traits and time-averaging effects of sampling methods, (2) to examine patterns in shell data that may contain a full seasonal cycle or more in medium-length and long shell profiles, and (3) to isolate short profiles, which only capture a season or less of growth and therefore would bias the inferred temperature record based on their season of collection.

Using subsampling methods and the directionality of sampling described in each paper, we expressed shell profiles in terms of “distance from growing margin” so that 0 mm represents the most recent shell material to precipitate, closest to the date of harvest or collection. Long profiles were analyzed to examine ontogenetic variability, identify seasonal isotopic trends, and assess variability among individuals. For long-profile modern shells, we compared seasonal interpretations with known instrumental SST records for relevant southern California sites. Both medium and long profiles (five or more subsamples containing multiple seasons of growth per shell) were used for geographic and millennial-scale analyses. Short profiles (< 5 subsamples per shell) were examined in comparison to long profiles and, whenever possible, in conjunction with the annual temperature cycle, to test whether *M*. *californianus* shells reliably recorded sub-seasonal δ^18^O-inferred temperature trends since short δ^18^O_shell_ profiles from archaeological molluscs are commonly used to infer season of harvest or site occupation [e.g., 82]. Short profiles were also used to investigate the influence of ontogenetic stage and tidal height on terminal edge δ^18^O_shell_ values. We compared δ^18^O_shell_ profiles of different lengths for shells of the same ^14^C age and from the same island to determine whether the length of the subsample profile impacts the interpretations of shell chemistry (S1 Fig). Lastly, we compared the mussel record to offshore marine sediment and foraminiferal isotopic records for the same time interval and region.

To analyze ontogenetic trends, local polynomial regression was applied to the longest δ^18^O_shell_ profile. We accessed seawater temperature data for 2000 through 2005 from the National Data Buoy Center (NDBC) Station 46054 (34.273° N, 120.470° W) and Point Dume Shore Station Data provided by the Shore Stations Program at Scripps Institute of Oceanography (34.014° N, 118.822° W) to compare the annual temperature cycle with the longest δ^18^O_shell_ record matching this same range of years. For statistical analysis, Pearson’s correlation was used to test relationships between ontogenetic stage (total shell length) and δ^18^O_shell_ and δ^13^C_shell_ values in short profiles whenever shell length data were available. Linear regression (Ordinary Least Squares, OLS) and Pearson’s correlation were used to evaluate intra-shell δ^18^O_shell_-δ^13^C_shell_ relationships in long-profile individuals. Analysis of variance (ANOVA) was applied to modern shells with medium and long profiles to test for differences of δ^18^O_shell_ and δ^13^C_shell_ values between island and mainland settings. ANOVA and Tukey HSD were also used to assess differences between individual sites and across tidal heights whenever tidal height data were available. Tukey HSD was used to determine which tidal heights were statistically different from one another in isotopic composition and evaluate the impacts of time-averaged subsampling. ANOVA and Tukey HSD were also used to determine whether δ^18^O_shell_ values were significantly different from one another over each millennium throughout the Holocene. A Welch Two-Sample t-test was used to compare modern and midden δ^13^C_shell_ values. All statistical analysis was performed in R [84].

### Paleotemperature and δ^18^O of seawater

To examine temperatures recorded by *M*. *californianus* through the Holocene, we applied the δ^18^O-SST equation developed by Epstein et al. (1953) [85] and calibrated for *M*. *californianus* by Killingley (1981) [86], where δ^18^O_w_ is the stable oxygen isotope value of seawater:

$$\text{SST}\left({}^{\circ}\text{C}\right)=16.4-4.2\left(\left(\delta^{18}{\text{O}}_{\text{shell}}-\delta^{18}{\text{O}}_{\text{w}}\right)\right)+0.13{\left(\left(\delta^{18}{\text{O}}_{\text{shell}}-\delta^{18}{\text{O}}_{\text{w}}\right)\right)}^{2}$$ (1)

The 13 studies included in our synthesis employed a variety of approaches for δ^18^O_w_. Multiple studies [72, 76, 80] used the modern measured δ^18^O_w_ value of -0.32‰ from a seawater sample collected by Rick et al. (2006) off the eastern coast of Santa Rosa Island [52]. Three different studies used the δ^18^O_w_ value of -0.32‰ but applied an ice-volume correction [53, 78, 79] for reconstruction of Holocene paleo-temperatures [87]. Two papers did not provide a δ^18^O_w_ value. Since sea level has been relatively constant since 6000 BP [88, 89], we applied an ice-volume correction to δ^18^O_w_ for shells with a radiocarbon age older than 6000 BP only and used the modern δ^18^O_w_ value of -0.32‰ for all other Channel Islands shells younger than 6000 BP. We used established relationships between sea-level change and δ^18^O_w_; a 10 m increase in sea level results in a 0.1‰ change in δ^18^O_w_ [90, 91]. Assuming a linear change in ice volume during the early Holocene, sea level rose 10 m per millennium [88, 89, 91]. Therefore, we used δ^18^O_w_ = -0.32‰ + 0.3‰ for mussel shells aged 9000–8000 BP, -0.32‰ + 0.2‰ for mussel shells aged 8000–7000 BP, and -0.32‰ + 0.1‰ for shells aged 7000–6000 BP (Table 2). While δ^18^O_w_ can vary on a seasonal scale in addition to a millennial scale, Jazwa et al. (2020) reported that the standard deviation of δ^18^O_w_ values from water samples collected seasonally at Bechers Pier on Santa Rosa Island from August 2015 to May 2019 was 0.1‰, ranging from a minimum of -0.6‰ to a maximum of -0.2‰ [74]. The low seasonal variance of δ^18^O_w_ would yield a temperature difference that is substantially lower than the seasonal temperature range of ~ 10°C, so we used the same modern δ^18^O_w_ value for all data points for which an ice-volume correction was not applicable.

**Table 2:** Treatment of δ^18^O_w_ values for this study according to binned age range (years BP).

Age range	Ice volume correction	δ^18^O_w_ value used	δ^18^O_shell_ data sources
9000–8000 BP	0.3‰	-0.02‰	Jew et al., 2013a; Jew et al., 2013b [78, 79]
8000–7000 BP	0.2‰	-0.12‰	Robbins et al., 2013 [80]
7000–6000 BP	0.1‰	-0.22‰	Robbins et al., 2013 [80]
6000 BP—0 BP	None; measured by Rick et al. (2006) [52]	-0.32‰	Glassow et al., 1994; Kennett, 1998; Rick et al., 2006; Ford et al., 2010; Glassow et al., 2012; Ferguson et al., 2013; Robbins et al., 2013; Jew and Rick, 2014; Jazwa and Kennett, 2016; Flores, 2017; Jazwa et al., 2020 [42, 45, 51–53, 72, 74, 76, 77, 80, 81]

Some papers spanned multiple millennia and are therefore listed more than once.

## Results and discussion

### Ontogenetic variability

#### Ontogenetic trends revealed by long profiles

Of all individual profiles analyzed here, the *M*. *californianus* shell with the longest stable isotope profile was collected at Cuyler Harbor, San Miguel Island in 2005 CE [80]. The shell was serially sub-sampled every 2 mm at 60 points along the ontogenetic growth trajectory, representing 118 mm of shell growth [80]. The length and resolution of this record presented the best opportunity to examine ontogenetic variation; the next longest individual shell record from the Channel Islands has 25 subsamples spanning 48 mm of growth.

**Fig 2:**
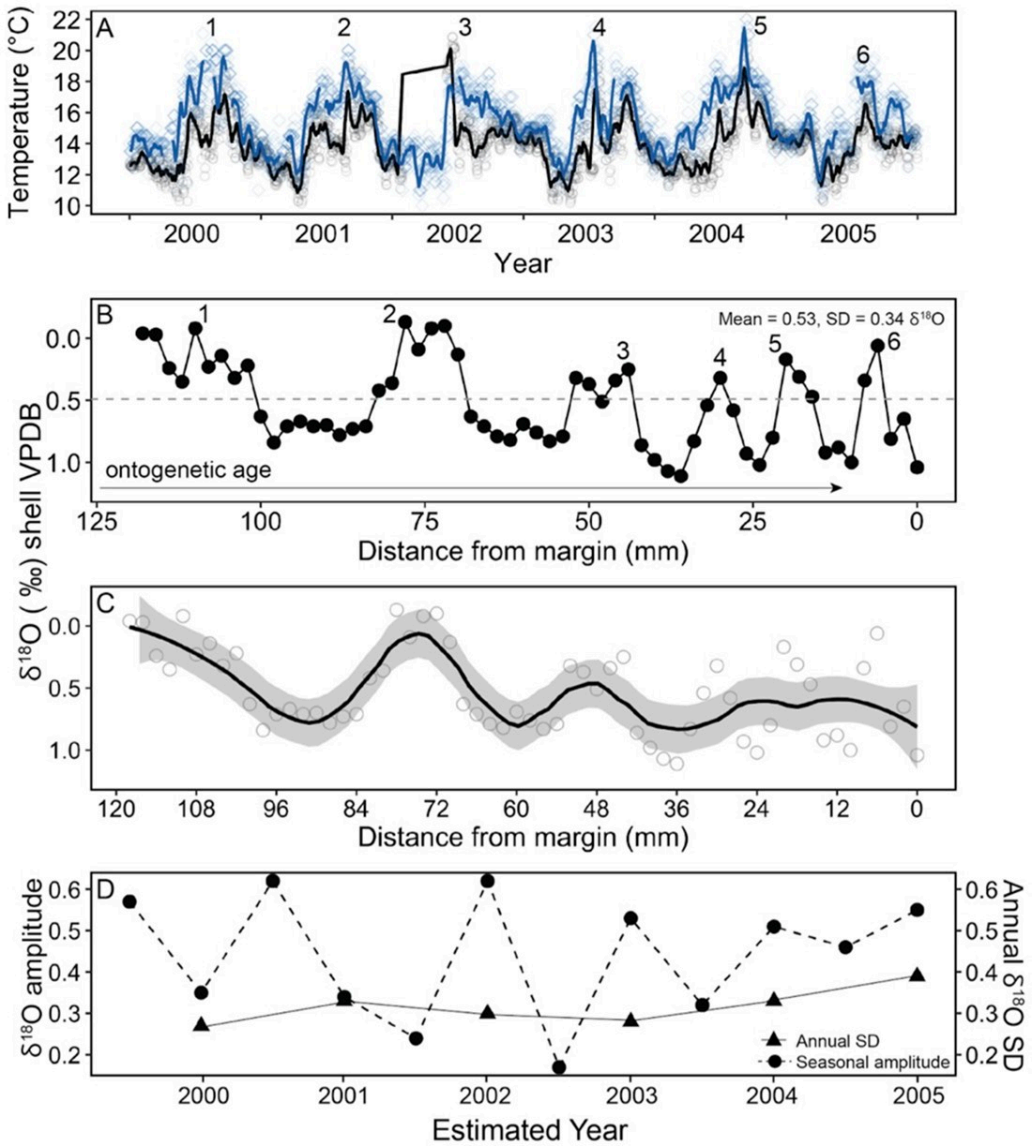
Stacked plot comparing seawater temperatures and the longest δ^18^O_shell_ profile. (A) Seawater temperatures for 2000–2005. Black line is a two-week running mean for NDBC Station 46054 in Santa Barbara Basin. Gray open circles are daily SST. Blue line is two-week running mean for Point Dume provided by the Shore Stations Program sponsored at Scripps Institution of Oceanography. Blue open circles are daily seawater temperatures. Numbers indicate maxima for each year of the record. (B) 118 mm of δ^18^O_shell_ from a *M*. *californianus* individual collected in 2005 at San Miguel Island. Gray dashed line represents the midpoint value of 0.49‰. Labeled numbers represent inferred warm seasons best matched with panel (A). (C) Local polynomial regression of δ^18^O_shell_ showing that modeled seasonal cycles become less apparent as the shell ages ontogenetically due to slower growth. Shaded region represents 95% confidence interval. (D) Calculated seasonal amplitude of δ^18^O_shell_ (dark circles and dashed line) and annual standard deviation of δ^18^O_shell_ (black triangles and solid line).

The San Miguel 2005 shell recorded an average δ^13^C_shell_ value of -0.51‰, an average δ^18^O_shell_ value of 0.53‰, and six identifiable annual cycles in its δ^18^O_shell_ profile (Fig 2). The δ^18^O_shell_ minima represent warm seasons recorded by the shell and match the two instrumental temperature records from 2000–2005 that we examined for comparison (Fig 2a): National Data Buoy Center (NDBC) Station 46054 located in the Santa Barbara Channel (34.273° N, 120.470° W) and Point Dume Shore Station Data provided by the Shore Stations Program at Scripps Institute of Oceanography (34.014° N, 118.822° W) [80]. Comparing annual temperature profiles to the δ^18^O_shell_ profile indicates that the amount of time represented by evenly spaced (2 mm) subsamples decreases as the shell ages (Fig 2a and 2b). In the years 2000 and 2001, earlier in the individual’s life, the subsamples represent sub-monthly resolution (i.e., ~ 14–18 subsamples capturing 12 months of growth). Following fast growth during earlier stages of life, growth reduction could be linked to the attainment of sexual maturity, when mussels reallocate a greater portion of energy from shell growth to gametogenesis [43, 92]. In the final two years of the individual’s life (2004 and 2005), one full annual cycle is captured within 6–7 subsamples (Fig 2b). This indicates that *M*. *californianus* slows its growth from ~ 2.5 mm per month earlier in its life to ~ 1 mm per month within a span of five years. Ontogenetic growth reduction amplifies the effect of time-averaging introduced by the sampling strategy, as shown by the less apparent annual cycles when local polynomial regression was applied to the δ^18^O_shell_ profile with evenly spaced samples (Fig 2c). When *M*. *californianus* is growing more slowly in later stages of ontogeny, more subsamples are required to capture environmental periodicity (e.g., temperature seasonality) since the individual is calcifying, and therefore recording, less environmental information in the same amount of time.

Since growth reduction throughout ontogeny has been well documented for accretionary carbonates [93–97], we predicted that the *M*. *californianus* δ^18^O ontogenetic profile from umbo to commissure would contain the following characteristics: (1) sinusoidal in shape, but increasingly cuspate as the individual aged, (2) a progressive decline in amplitude, (3) a progressive increase in frequency, and (4) a progressive decline in wavelength. As predicted, the δ^18^O_shell_ profile was generally sinusoidal with sharp cusps marking seasonal patterns during the last three years of its life (Fig 2b). The increasingly cuspate shape of the δ^18^O local minima suggests that growth slowdown occurred as the organism aged, resulting in shorter seasonal cycles [97–99]. Generally, the frequency increased, and the wavelength declined as predicted. Unexpectedly, however, this individual’s δ^18^O_shell_ profile did not exhibit an ontogenetic decline in seasonal amplitude (Fig 2d), as determined by calculating the difference between the local δ^18^O_shell_ extrema for each warm or cool season and the profile’s midpoint δ^18^O_shell_ value (0.49‰). This results in six warm and six cool seasons over the six-year period recorded by this individual. If δ^18^O_shell_ values lower than the midpoint value of 0.49‰ are considered to be warm seasons and δ^18^O_shell_ values higher than this are interpreted as cool seasons, then the shell recorded warm seasons for 41.7% and cool seasons for 58.3% of the 118 mm-long shell record. More fully profiled individuals of this size would help determine whether these percentages reflect a true environmental signal driven by the annual temperature cycle, or whether they are biologically mediated due to growth shutdown/slowdown in warmer conditions, particularly as the organism aged. Throughout ontogeny, the shell continued to record seasonal cyclicity but recorded less sub-seasonal variability (Fig 2b). Additional closely spaced subsamples towards the terminal edge of the shell would likely help capture finer-scale variability (e.g., weekly to monthly) in an aging individual.

We also found a small overall increase in annual standard deviation of δ^18^O_shell_ from 0.27‰ in 2000 to 0.39‰ in 2005 (Fig 2d). This increase was expected since a lower standard deviation earlier in life represents the lower short-term variation between temporally close samples, and a higher standard deviation later in life reflects the greater variability or larger temperature swings from one subsample to the next due to more temporal gaps in the calcified record, and therefore fewer subsamples representing a season of growth.

**Fig 3:**
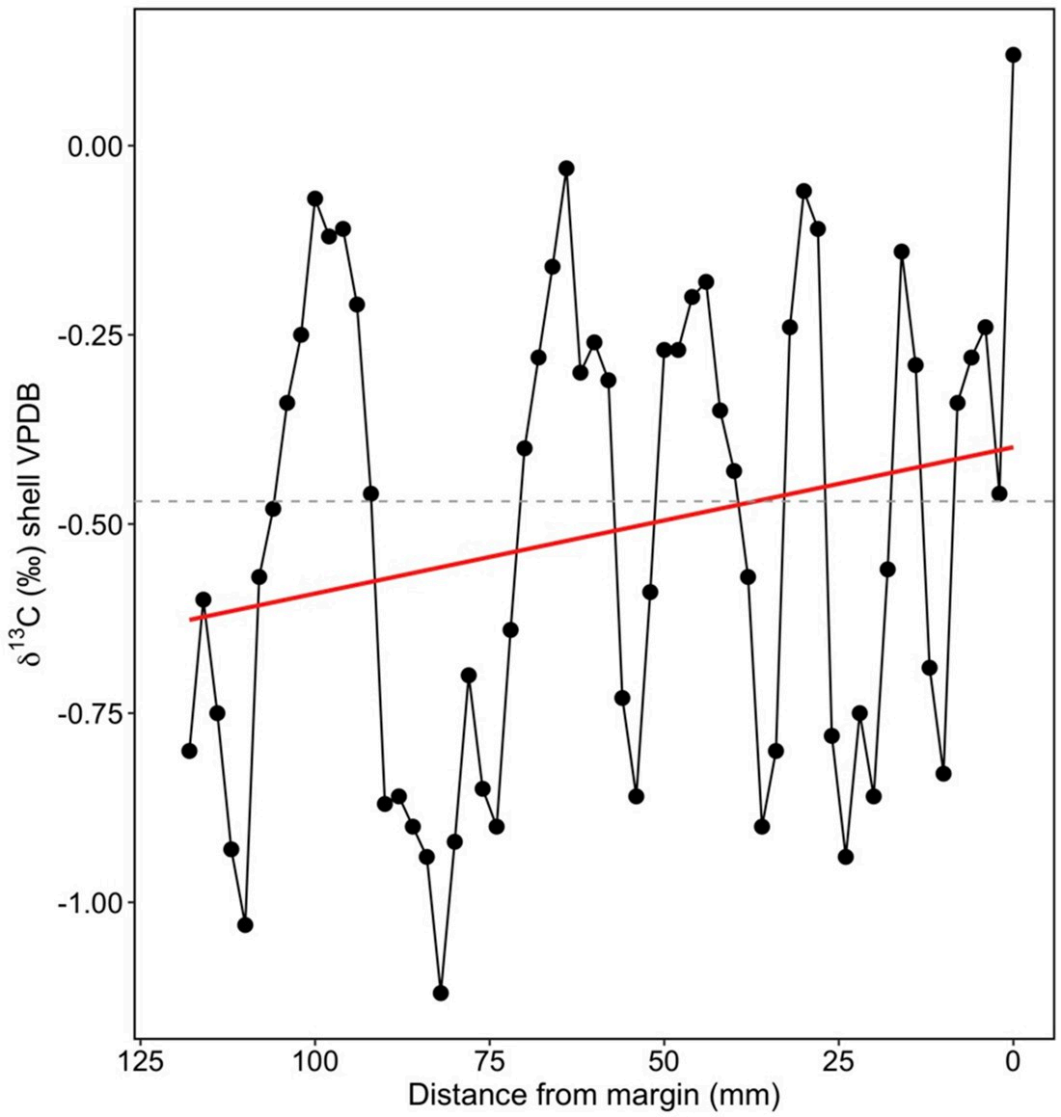
δ^13^C_shell_ profile of San Miguel Island *M*. *californianus* individual collected in 2005. X-axis represents ontogenetic growth from left to right (e.g., 0 mm is closest to time of death). Gray dashed line represents a median δ^13^C_shell_ value of -0.47‰. Regression line (in red) indicates ontogenetic trend towards positive δ^13^C_shell_ values.

The ontogenetic δ^13^C_shell_ profile of the same individual from San Miguel Island was examined separately since δ^13^C is a more complex proxy than δ^18^O in biogenic carbonates due to the varying impacts of ocean circulation, upwelling, primary productivity, seawater DIC δ^13^C, and metabolic effects on δ^13^C_shell_ profiles [45]. In this individual, the ontogenetic δ^13^C_shell_ profile records six annual cycles with increasing frequency (Fig 3), which could reflect seasonal upwelling/relaxation oscillations in the Santa Barbara Channel. There was an ontogenetic trend towards more positive δ^13^C_shell_ values (Fig 3), which does not fit the prediction that the organism incorporates a greater amount of isotopically light metabolic carbon as it ages, as found in other marine mussel species including *Pinna nobilis* and *Mytilus edulis* [100–102]. The unexpectedly positive δ^13^C_shell_ trend found in this individual could be associated with ontogenetic growth reduction in this species. Slower shell growth and lower metabolic rates could result in reduced incorporation of respired carbon and therefore higher δ^13^C_shell_ values with age [102]. Alternatively, the positive δ^13^C_shell_ trend could be indicative of changes in the timing of growth as the organism aged, such as less growth during periods of upwelling (low δ^13^C) throughout ontogeny.

#### Ontogenetic trends in short profiles

Since previous studies of other mussel species identified ontogenetic de-coupling between environmental parameters and shell chemistry [100, 101], we tested the relationship between ontogenetic stage (shell length) and growing edge δ^18^O_shell_ and δ^13^C_shell_ values in 76 *M*. *californianus* shells live-collected on the same day at Santa Rosa Island in August 2017 [74]. These 76 individuals were chosen for this analysis because this was the only dataset to include shell length information for each individual. Shell length cannot be assumed from subsampling distance (e.g., 10 mm of shell subsamples could be from an individual of any shell length longer than 10 mm). The primary authors sub-sampled each of these 76 individuals at three points spaced 1 mm apart at the terminal edge of the shell and calculated a mean δ^18^O_shell_ and mean δ^13^C_shell_ value for the final 3 mm of growth for each shell [74]. These mean terminal edge values are time-averaged, aiming to represent ~1–3 months of growth depending on the ontogenetic age and growth rate of the shell. Shell lengths ranged from 42.5 mm to 118.5 mm with a mean of 73.9 mm. The δ^18^O_shell_ values ranged from -1.12‰ to 1.17‰, and δ^13^C_shell_ values ranged from -1.56‰ to 0.34‰. In our analysis of these 76 individuals, we found no significant relationship between shell length and mean terminal edge δ^18^O_shell_ (Pearson’s correlation test, t = -1.5613, df = 74, p = 0.1227). Similarly, there was no significant relationship between shell length and mean terminal edge δ^13^C_shell_ (Pearson’s correlation test, t = 1.5254, df = 74, p = 0.1314). However, the lack of relationship between shell length and end-of-life δ^18^O_shell_ and δ^13^C_shell_ values is not necessarily an indication that ontogenetic patterns do not strongly influence shell chemistry; we found that ontogenetic stable isotope profiles show a marked increase in frequency and a decrease in wavelength with age (Figs 2 and 3). There could also be site-dependent influences on ontogenetic effects at the localities where the specimens were collected. Longer shell profiles capturing more growth are required to disentangle ontogenetic effects and reconstruct environmental cycles at a variety of sites.

### Geographic variability

#### Geographic trends in modern shells

**Fig 4:**
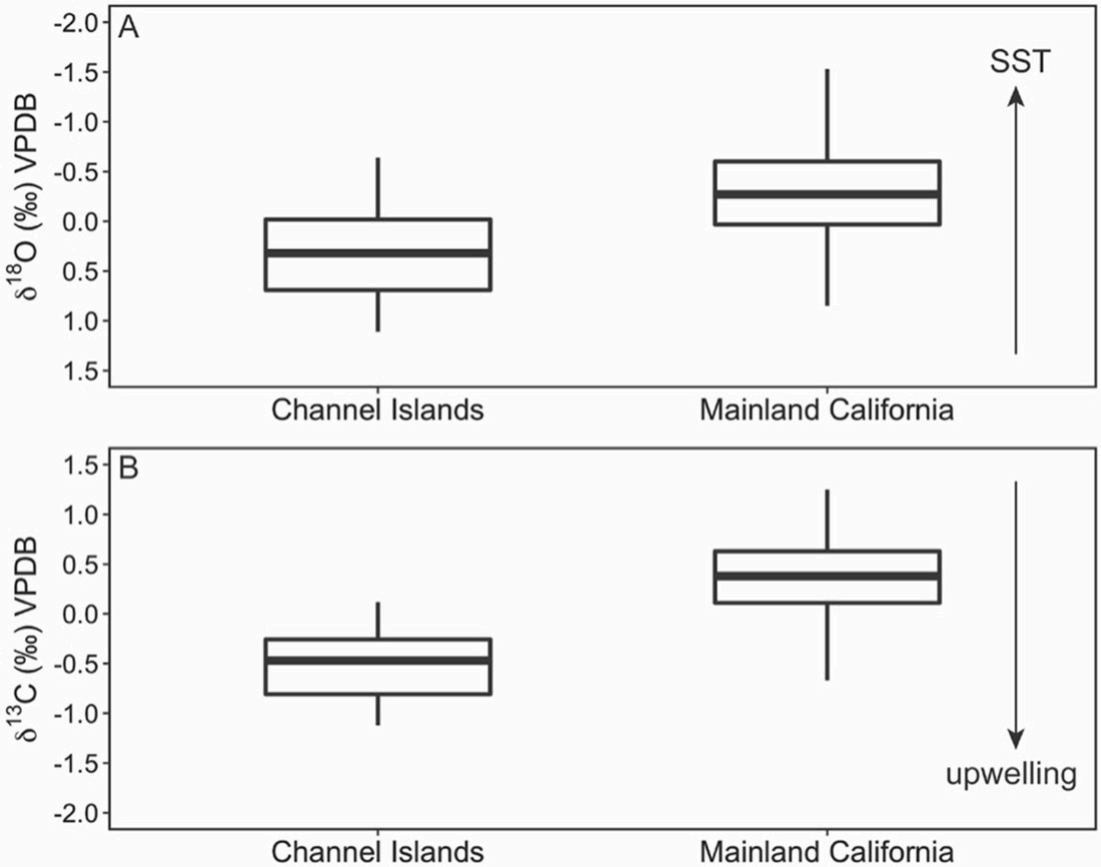
Box plots showing stable isotopic ranges of all modern shells by location. Channel Islands includes all data points from all modern shells with five or more subsamples from San Miguel Island and Santa Cruz Island. (There were no modern shells with more than five subsamples from Anacapa or Santa Rosa Island.) Mainland California includes all data points from all modern shells with five or more subsamples from San Diego and Newport Beach. Black bar is the median value. (A) Oxygen isotope range of modern δ^18^O_shell_ in Channel Islands vs. mainland shells. (B) Carbon isotope range of modern δ^13^C_shell_ in Channel Islands vs. mainland shells. Arrow shows expected directionality of a potential δ^13^C upwelling signal.

Analysis of modern shells with five or more subsamples (medium and long profiles) revealed a significant difference in shell chemistry between *M*. *californianus* shells collected from the Channel Islands versus mainland southern California (Fig 4, ANOVA, oxygen: F_1,735_ = 194.1, p < 0.001, carbon: F_1,679_ = 318.2, p < 0.001). In present-day conditions, weak wind forcing and the delivery of warm water by the northbound California Undercurrent and Davidson Current produce a significantly warmer regime along the Southern California Bight than farther offshore (Fig 1), and this pattern is reflected in statistically different *M*. *californianus* shell chemistry: lower δ^18^O_shell_ values (mean ± 1σ = -0.32‰ ± 0.47‰) from the mainland sites (Newport Beach and San Diego) relative to higher δ^18^O_shell_ values (mean ± 1σ = 0.33‰ ± 0.42‰) in the Channel Islands shells (Fig 4a). Additionally, the lower δ^13^C values of Channel Islands shells (Fig 4b) may reflect the islands’ proximity and exposure to the biologically productive California Current. The south-facing coastline of the Southern California Bight is protected from intense wind forcing [46], resulting in weaker upwelling. Weaker upwelling is typically associated with higher δ^13^C_shell_ values, as found in the mainland shells relative to δ^13^C_shell_ from the offshore islands (Fig 4b).

#### Geographic variability through time

**Fig 5:**
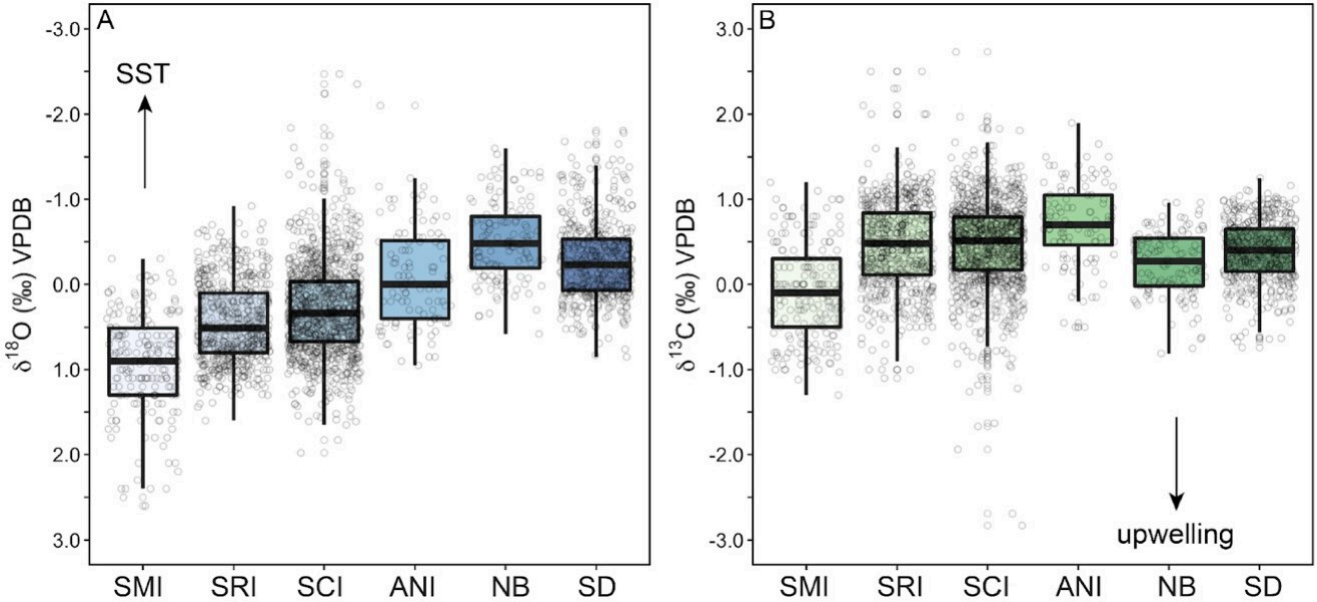
Box plots showing stable isotope ranges of each site comprising all shells with five or more subsamples from all Holocene intervals. Black bar is the median value. (A) Oxygen isotope data from all shells with five or more subsamples for San Miguel Island (SMI), Santa Rosa Island (SRI), Santa Cruz Island (SCI), Anacapa Island (ANI), Newport Beach (NB), and San Diego (SD). Y-axis is inverted to match directionality of temperature proxy as indicated by SST arrow. (B) Carbon isotope data from all shells with five or more subsamples for the same sites. Arrow shows expected directionality of a potential δ^13^C upwelling signal.

Oxygen and carbon isotope data from all shells with five or more subsamples (medium and long profiles) were used to calculate summary statistics for each site (San Miguel Island, Santa Rosa Island, Santa Cruz Island, Anacapa Island, Newport Beach, and San Diego) across all time intervals as shown in Table 3. Throughout the study period, *M*. *californianus* collectively recorded warmer SSTs from west to east, with stepwise depletion in median δ^18^O_shell_ from San Miguel Island to Anacapa Island (Fig 5a). Differences in δ^18^O_shell_ by site were statistically significant (ANOVA, F_5,2658_ = 252.7, p < 0.001). Tukey HSD revealed significant differences between all sites in medium and long δ^18^O_shell_ profiles across the study interval (S1 Table). Overall, mussels from Newport Beach and San Diego recorded the warmest δ^18^O_shell_ signals. Despite most of its shell samples originating within the late Holocene (since 4200 BP), Santa Cruz Island had the widest range of δ^18^O_shell_ values, which is likely a product of its location and oceanographic setting. The California Current delivers cool water to western Santa Cruz Island, while SSTs on the eastern portion of the island are influenced by the warmer Davidson Current and Southern California Countercurrent. The dynamic nature of multiple oceanographic currents at this site may explain greater δ^18^O_shell_ intra-island variability for Santa Cruz Island mussels [72].

**Table 3:** Summary statistics for all shells with five or more subsamples, sorted by site.

Site	Mean δ^18^O	Min δ^18^O	Max δ^18^O	σ δ^18^O	Mean δ^13^C	Min δ^13^C	Max δ^13^C	σ δ^13^C	Total number of subsamples
San Miguel Island	0.91	-0.3	2.6	0.58	-0.08	-1.3	1.2	0.55	197
Santa Rosa Island	0.45	-0.92	1.6	0.47	0.47	-1.1	2.5	0.51	612
Santa Cruz Island	0.29	-2.47	1.98	0.55	0.46	-2.83	2.73	0.52	1072
Anacapa Island	-0.1	-2.1	0.95	0.58	0.71	-0.5	1.9	0.48	183
Newport Beach	-0.5	-1.6	0.58	0.45	0.24	-0.81	0.96	0.38	125
San Diego	-0.27	-1.81	0.85	0.46	0.39	-0.74	1.25	0.36	499

There is an east-west δ^13^C_shell_ gradient among the Channel Islands shells. Farthest offshore, San Miguel Island mussels record the lowest δ^13^C_shell_ values, and overall δ^13^C_shell_ values increase towards the mainland, with Anacapa Island mussels recording the highest δ^13^C_shell_ values (Fig 5b). Newport Beach and San Diego mussel shells were isotopically lighter than shells from the eastern Channel Islands, but since the mainland δ^13^C_shell_ values represent modern mussels only (live-collected in the 2000s) and the island δ^13^C_shell_ values represent all time points throughout the Holocene, this offset may be a temporal trend rather than geographic. Anthropogenic burning of fossil fuels has changed atmospheric chemistry, and in turn, lowered the δ^13^C composition of seawater DIC and marine biogenic carbonates [58, and references therein]. Additionally, recent increases in upwelling strength and greater nearshore productivity have been documented along coastal mainland California [103], which may also be reflected in the lighter δ^13^C_shell_ composition of the modern mainland shells. As expected, modern shells were collectively depleted relative to the midden shells (Welch Two Sample t-test, t = -18.589, df = 2010.5, p < 0.0001). Our dataset contains no modern δ^13^C_shell_ values from Santa Cruz Island or Anacapa Island, but when comparing δ^13^C_shell_ of modern shells only, Santa Rosa and San Miguel Island shells maintained lower δ^13^C_shell_ values than the modern mainland shells. While significant terrestrial influences, such as plant decomposition, river input, or agricultural runoff, would likely result in isotopically lighter carbon rather than the higher mainland δ^13^C_shell_ values found here, it is also possible that the collective analysis of aggregated δ^13^C_shell_ values obscures the influences of terrestrial and freshwater systems. Collectively, δ^13^C_shell_ in intertidal mussels may serve as a better indicator of relative oceanographic conditions driven by δ^13^C of the DIC across a geographic region rather than as an indicator of individual events (e.g., a freshwater input pulse due to a storm). The reliability of δ^13^C_shell_ as an environmental proxy is limited due to the significant and complex role of metabolic δ^13^C incorporation into the shell as well as the competing environmental influences of upwelling, productivity, salinity, freshwater input, and the Suess Effect [56, 58].

#### Geographic variability by millennium

**Fig 6:**
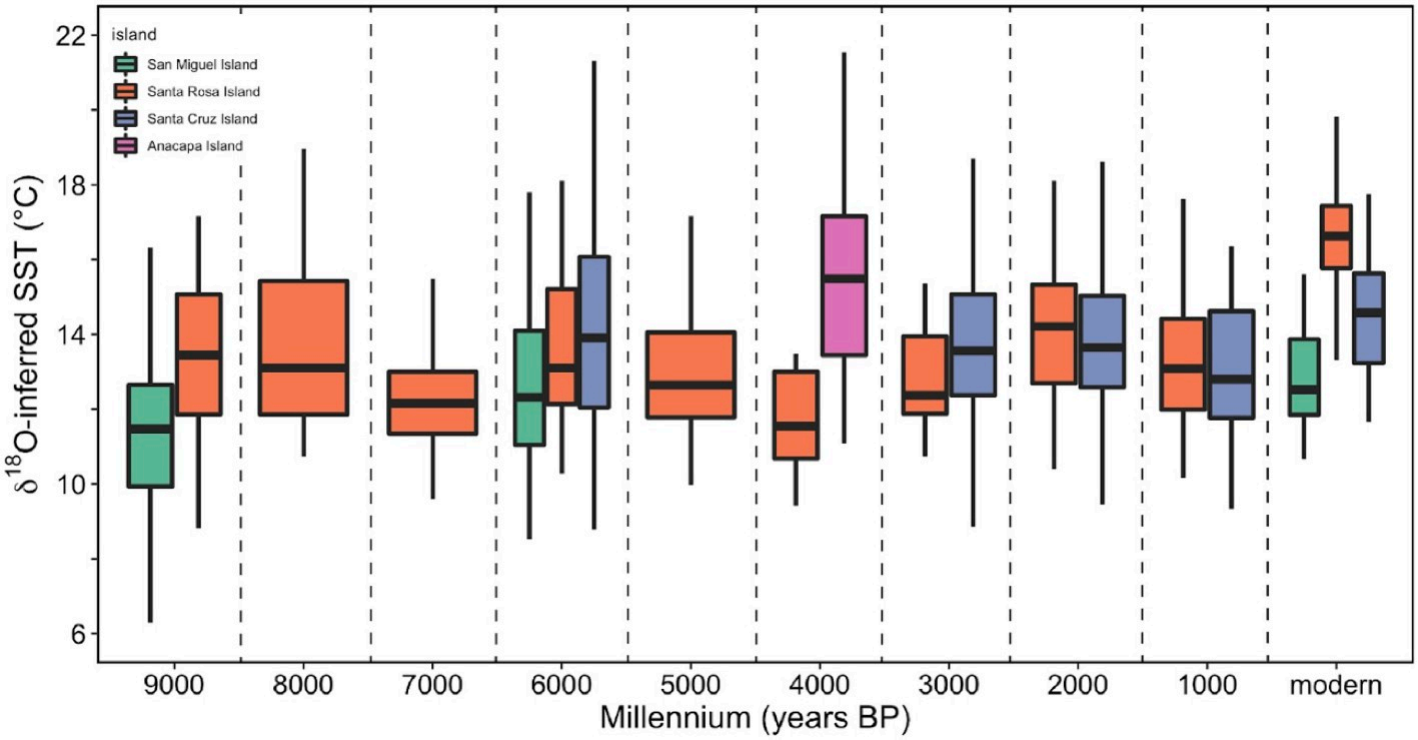
Millennial scale δ^18^O_shell_-inferred temperature variability for each island. Data were binned by millennium for each island to produce a range of temperature snapshots. Black bar is the median value.

We binned oxygen isotope data from shells with five or more subsamples (medium and long profiles) by millennium for each island and converted these values to SST (°C) using Eq 1 to produce a record of temperature snapshots throughout the Holocene. Santa Rosa Island offered the most temporally continuous record with data points spanning every millennium from 9000 BP through the present (Fig 6). Temperatures recorded at Santa Rosa Island were highest (> 14°C) between 9000–8000 BP and lowest (11.67°C) between 4000–3000 BP (Table 4). Both the geologically oldest and youngest shells were from San Miguel Island (8800 BP and 2005 CE), allowing for a comparison between two snapshots at both ends of the record. Inferred San Miguel mean SST was low (11.99°C) during the early Holocene and was overall higher in 2005 CE (12.92°C), although these differences are based on uneven distributions of data and were not statistically significant. Anacapa Island had only one snapshot at 3010 BP but recorded a wide range of temperatures (11.08° to 24.76°C; Table 4). Oxygen isotope data from the three westernmost islands (San Miguel Island, Santa Rosa Island, and Santa Cruz Island) within 6000–5000 BP preserved the geographic east-west SST gradient that characterizes the northern Channel Islands today, indicating that mussels did not collectively record fine-scale changes in California Current and sub-current forcing at 6000 BP that other models and records have documented [104]. The late Holocene is well represented at Santa Cruz Island with snapshots from the past three millennia. Calculated mean temperatures for each millennium at Santa Cruz Island exhibit warm-cool millennial scale variability: 14.13°C during 6000–5000 BP, 13.77°C during 3000–2000 BP, 14.23°C during 2000–1000 BP, 13.05° in midden shells from the past 1000 years, and 14.6°C in modern shells (Table 4). Temperature variability was highest at Santa Cruz Island within 2000–1000 BP (σ = 2.71°C) and lowest at Santa Rosa Island within 3000–2000 BP (σ = 1.34°C). Shells from Santa Cruz and Anacapa Islands recorded the highest overall maximum inferred temperatures, all three of which occurred in the late Holocene (Table 4). San Miguel Island reported the lowest overall minimum inferred temperature (6.29°C) at 8800 BP.

**Table 4:** Summary statistics for all shells with five or more subsamples, sorted by both island and millennium.

Island	Millennium	Mean δ^18^O	σ δ^18^O	Mean SST	σ SST	Max SST	Min SST
San Miguel Island	9000 BP	1.08	0.59	11.99	2.29	15.6	6.29
	modern	0.53	0.34	12.92	1.37	12.5	10.66
Santa Rosa Island	9000 BP	0.41	0.48	14.64	1.94	18.45	9.94
	8000 BP	0.36	0.56	14.47	2.28	19.84	11.51
	7000 BP	0.67	0.36	12.78	1.46	16.78	9.97
	6000 BP	0.36	0.49	13.62	1.98	18.1	10.28
	5000 BP	0.52	0.42	13	1.68	17.16	9.98
	4000 BP	0.85	0.36	11.67	1.39	13.48	9.41
	3000 BP	0.55	0.38	12.85	1.34	15.35	10.74
	2000 BP	0.25	0.43	14.06	1.74	18.1	10.39
	1000 BP	0.43	0.5	13.36	2.05	17.63	10.16
Santa Cruz Island	6000 BP	0.24	0.63	14.13	2.57	21.31	8.78
	3000 BP	0.33	0.5	13.77	2.03	24.94	7.43
	2000 BP	0.22	0.65	14.23	2.71	26.03	8.63
	1000 BP	0.5	0.44	13.05	1.74	16.36	9.34
	modern	0.12	0.38	14.6	1.57	17.75	11.66
Anacapa Island	4000 BP	-0.19	0.6	15.6	2.52	24.76	11.0

Each millennium includes all samples within that millennium (e.g., 1000 represents all non-modern shells from 0–1000 BP, etc.). SST was calculated by applying the equation from Epstein et al. (1953) modified for *M*. *californianus* by Killingley (1981) [85, 86]. δ^18^O is reported in ‰ and SST is in °C.

### Seasonal variability

#### Seasonality revealed by medium and long profiles

**Fig 7:**
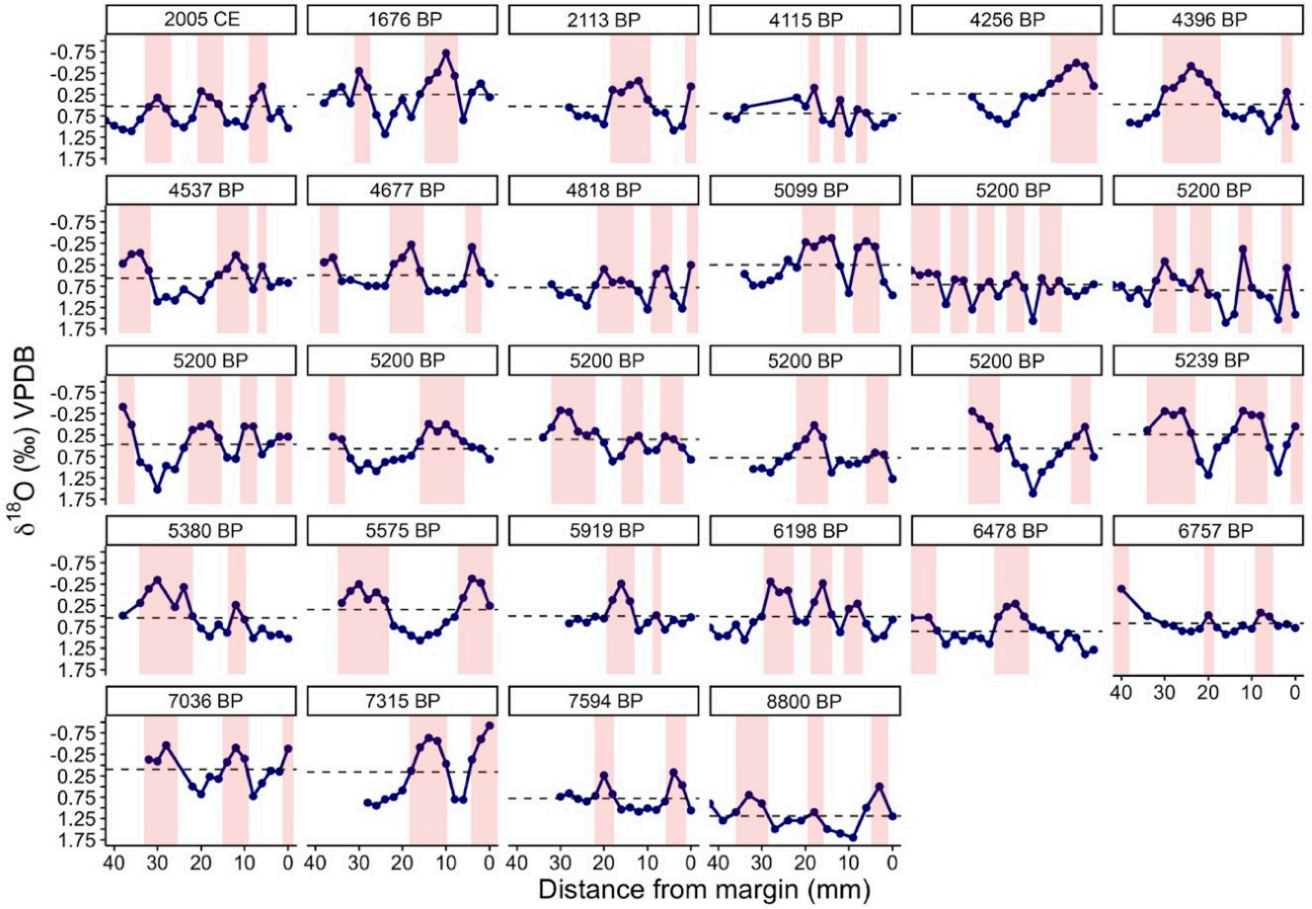
Individual oxygen isotope profiles of all 28 long profiles (15+ subsamples) from Channel Islands shells. Each profile is from a unique shell with collection year or calibrated age in years before present (BP) labeled at the top of the plot. Dashed line represents the mean oxygen isotope value recorded by each individual shell. Red shaded bars are estimations of warm seasons. Y-axis is inverted and the scale is uniform for all plots.

Shells with long profiles (15 or more subsamples per individual) were used to examine seasonal oscillations. Long oxygen isotope profiles were truncated to the final 40 mm to compare profiles of similar lengths. These profiles exhibited sinusoidal seasonal variability, with some individuals recording more pronounced seasonal extremes than others (Fig 7). Long-profile individuals (n = 28) ranged in geologic age from 8800 BP through 2005 CE, yet all truncated 40-mm profiles recorded at least one annual cycle with a mean of 2.6 ± 0.83 (1σ) warm seasons. Estimating the number of warm seasons serves as a way to estimate the age of an individual at the time of its death if the subsampling profile captures the full ontogenetic history of the shell. However, since the shells analyzed here are primarily archaeological shells harvested by humans, the inferred number of warm seasons indicates the age at which shells were typically harvested for consumption rather than the average lifespan of a mussel. The full potential lifespan of *M*. *californianus* is unknown but may range from 10 to as many as 100 years [31].

On average, the 28 long-profile individuals recorded a calculated SST range of 5.4°C (δ^18^O_shell_ = 1.35‰), which matches the typical annual temperature range for nearshore waters surrounding the Channel Islands [54, 74]. The highest SST was recorded by an individual at 7315 BP. The warmer inferred conditions are consistent with the oxygen isotope record of *G*. *bulloides* planktic foraminifera at ODP Site 893 A/B in Santa Barbara Basin for this same age range [105]. All *M*. *californianus*-inferred temperatures lower than 9.5°C were recorded between 5200 and 5099 BP, which was unexpected since this interval is considered to be a warm period for this region [105]. Local-scale changes in ocean circulation may have played a role in this misalignment, particularly since small-scale geographic variability appears to have an influence on shell chemistry (Fig 5).

**Fig 8:**
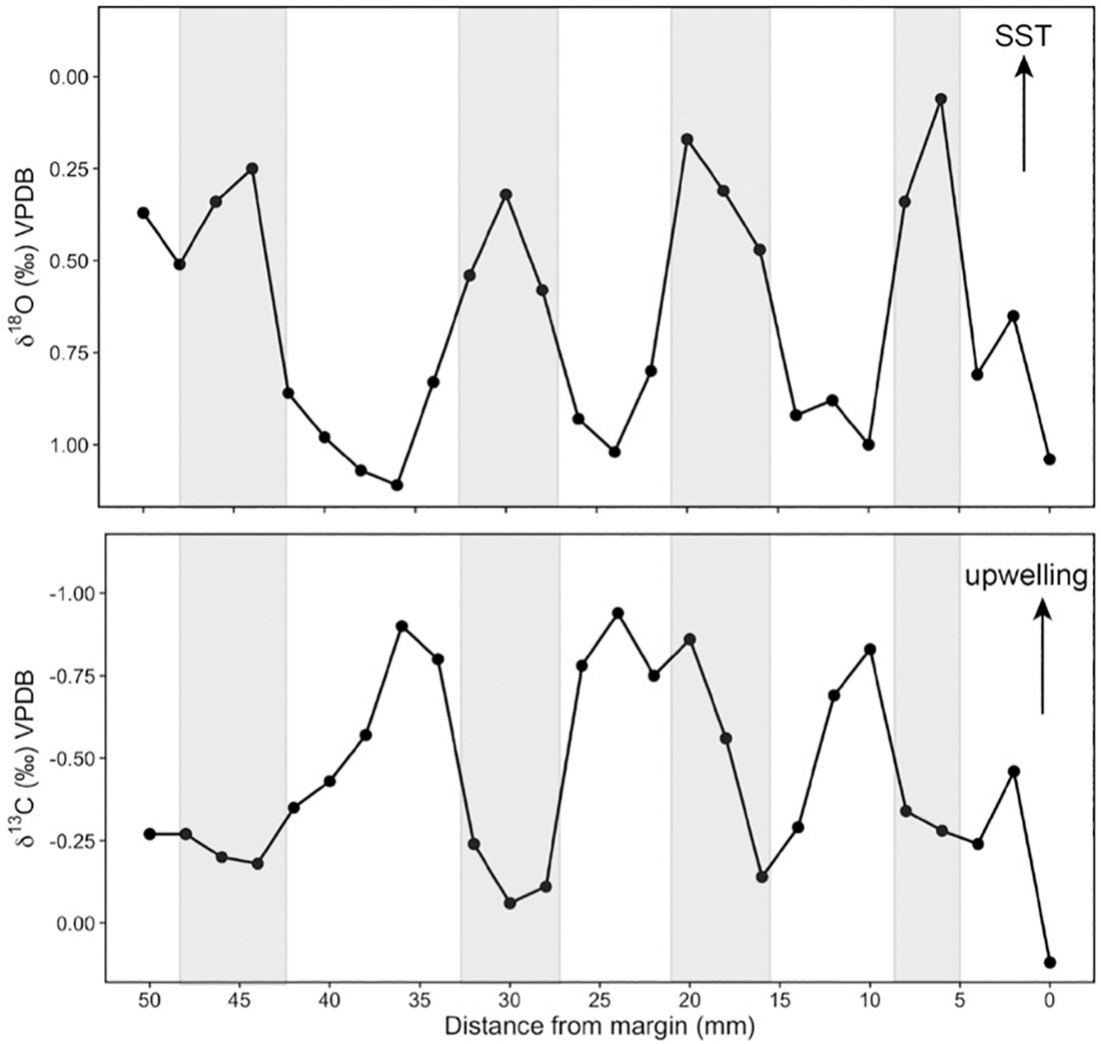
The last 50 mm of δ^18^O and δ^13^C profiles in a *M*. *californianus* shell collected from Santa Miguel Island in 2005 CE. In the top plot (δ^18^O), y-axis is inverted to match directionality of temperature proxy as indicated by SST arrow. Gray bars in both plots denote δ^18^O-inferred warm seasons. In the lower plot, arrow shows expected directionality of a potential δ^13^C upwelling signal.

Seasonality of carbon isotope profiles is less distinguishable. Out of all 28 long-profile shells, only one individual showed clear seasonal cycles that closely aligned with its oxygen isotope seasonal oscillations, and only a few individuals exhibited generally covarying δ^18^O_shell_ and δ^13^C_shell_ profiles (S2 Fig). While δ^13^C_shell_ profiles alone are challenging to interpret, strong correlation between δ^18^O_shell_ and δ^13^C_shell_ can be used to distinguish between the influences of rainfall and upwelling; a positive δ^18^O-δ^13^C relationship is indicative of freshwater input, while δ^18^O and δ^13^C are negatively correlated during upwelling [106, 107]. The individual with strongly expressed seasonal δ^13^C_shell_ cycles was collected in 2005 CE from San Miguel Island (Fig 2). In this individual, the oxygen isotope minima (inferred warm seasons) aligned remarkably well with carbon isotope minima (inferred low upwelling), particularly in the last 50 mm of the record (Fig 8). The strong seasonal δ^13^C_shell_ cycles in this individual are comparable to those found by Killingley and Berger (1979) in *M*. *californianus* shells from San Diego [59]. However, the vast majority (27 out of 28 individuals) lacked δ^13^C_shell_ profiles that aligned with seasonal interpretations of δ^18^O_shell_ cycles, and/or exhibited statistically insignificant relationships between δ^18^O_shell_ and δ^13^C_shell_. Similarly, Ferguson et al. (2013) found that δ^13^C_shell_ in southern California mussels could not be used to reliably reconstruct upwelling, likely due to metabolic carbon contributions to δ^13^C_shell_ relative to δ^13^C of DIC [45].

We performed OLS regression to investigate whether shells with a particular range of δ^18^O_shell_ values also contained a particular range of δ^13^C_shell_ variability [108], which could help distinguish between an upwelling signal (high δ^18^O_shell_, low δ^13^C_shell_) and a freshwater input (high δ^18^O_shell_, low δ^13^C_shell_) signal [106–107]. Only three of the 28 long-profile individuals analyzed here exhibited an inferred upwelling signal, or a statistically significant intra-individual negative δ^18^O_shell_-δ^13^C_shell_ correlation (Shells L, E, and Z in S2 Fig; Shell L: OLS linear regression, R^2^ = 0.67, F_1,13_ = 26.86, p < 0.001, Pearson’s correlation test, t = -5.1828, df = 13, p < 0.001; Shell E: OLS linear regression, R^2^ = 0.51, F_1,15_ = 15.7, p = 0.001, Pearson’s correlation test, t = -3.9622, df = 15, p = 0.001; Shell Z: OLS linear regression, R^2^ = 0.38, F_1,13_ = 8.118, p = 0.01, Pearson’s correlation test, t = -2.8492, df = 13, p = 0.01) and none of the individuals contained statistically significant positive δ^18^O_shell_-δ^13^C_shell_ relationships. The absence of a consistent upwelling signal across multiple individuals could be due to the input of metabolic carbon or the result of growth cessation during upwelling conditions. The three individuals that shared statistically significant negative δ^18^O_shell_-δ^13^C_shell_ relationships (Shells L, E, and Z in S2 Fig) have different ^14^C ages (7315 BP, 5200 BP, and 4256 BP, respectively) and are from different islands (Santa Rosa Island and Santa Cruz Island). The lack of consistent δ^18^O_shell_-δ^13^C_shell_ relationships among mussels from a particular site or time interval is likely influenced by sampling strategy, calcification rate, metabolic activity, and variability in micro-environmental conditions across the dynamic intertidal zone. High-resolution isotope sampling within this species and analysis of additional species from the same and adjacent environments are required to determine which factors influence δ^18^O_shell_-δ^13^C_shell_ relationships in molluscan carbonate and obscure upwelling signals.

While seasonality is visually apparent in the long δ^18^O_shell_ profiles (Fig 7), the number of subsamples required to capture seasonal variability depends on the growth rate, and therefore ontogenetic age, of the individual. Typically, ~ 3 mm is thought to represent 1–2 months of growth, and 8–10 closely spaced subsamples are required to identify an annual cycle [51, 74]. Although evenly spaced 2–3 mm increments is a conventional subsampling approach based on field observations of California mussels, we emphasize the need for consistently updated, long-term, and site-specific field studies of *M*. *californianus* shell growth rates because this species exhibits highly variable growth rates across sites and through time [29, 40, 44]. Since we observed that profiles with five subsamples contained a local minimum and a local maximum in most of our oxygen isotope profiles (i.e., interpreted as a warm season and a cool season, respectively), we used all individuals with five or more subsamples to infer trends over broader spatial and temporal scales (Figs 4–6). This greatly expanded our sample sizes (both n = number of individual shells and n = number of subsamples) and allowed for additional temporal snapshots and geographic comparisons. Individuals with fewer than five subsamples were excluded from broader scale interpretations since these short, end-of-life profiles capture only a season or less of growth, which could skew a long-term record. For example, if an individual collected in late summer is sampled at three evenly spaced points at its growing margin, its subsamples would likely only capture summer warming. This individual would introduce bias into an aggregated long-term record by indicating that conditions were warmer during its lifespan, so it is important to consider the time of year and length of time represented by the sampling method and interpret shell profiles accordingly.

#### Seasonality obscured by short profiles

Since terminal growth band (TGB) sampling is a sampling method commonly used to reconstruct the season of harvest from archaeological shells, we assessed whether short shell profiles (fewer than five subsamples) reliably captured sub-seasonal temperature trends (e.g., summer warming or winter cooling). Typically, ~ 3 mm of shell is thought to represent 1–2 months of growth in adult *M*. *californianus* shells from southern California, so a short profile at the terminal growing edge of the shell is often used to infer the time of year that the shell was collected [25, 29, 34, 74, 77, 82]. If *M*. *californianus* shells reliably captured sub-seasonal (monthly) temperatures at this sampling distance and resolution, a ~ 3 mm-long δ^18^O_shell_ profile with decreasing values would represent rising temperatures (spring-summer) while an increasing δ^18^O_shell_ profile of the same length would indicate falling temperatures (fall-winter). To test this, we selected the modern short shell profiles in our dataset with the same known collection date in August 2017 (n = 76 shells) at Santa Rosa Island. Out of these 76 live-collected individuals, only 48 recorded overall summer warming in the 3-mm long δ^18^O_shell_ profile at the terminal margin [74] (S3 Fig). These individuals spanned a variety of ontogenetic ages as implied by the range in shell length (42.5 mm to 118.5 mm) and were collected at various tidal heights (0 m to 1 m) at 10 cm increments on the same day [74]. The original authors reported a statistically significant negative correlation between δ^18^O_shell_ and water depth; the subsampled δ^18^O_shell_ values from the terminal edge of the shell decreased as water depth increased [74]. To test the effects of time-averaged sampling on this relationship, we used the mean δ^18^O_shell_ value from the 3-mm profile for each individual. We assigned tidal height categories to the water depth measurements provided by the authors: 0 to 30 cm is high, 30 to 70 cm is middle, and 70 to 100 cm is low tidal position. We found a significant difference in mean terminal δ^18^O_shell_ related to tidal height (ANOVA, df = 2, F-value = 12.29, p = < 0.01). There were statistically different mean terminal δ^18^O_shell_ values among mussels collected from the low and high tidal positions (Tukey HSD, p = < 0.001) and middle and high tidal positions (Tukey HSD, p = 0.03), but not among middle and low tidal positions (Tukey HSD, mid-low p = 0.13). Both individual subsamples [74] and time-averaged terminal-edge mean values revealed differences in δ^18^O_shell_ related to tidal height. These relationships are likely due to submergence times and aerial exposure; mussels in the high tidal position are submerged for less time, and therefore experience more physiological stress and growth interruption than mussels in lower tidal positions [43, 44, 74]. Oxygen isotope variability on a sub-seasonal scale may be less related to temperature and instead more closely tied to ontogenetic age, growth rate, tidal height, and the dynamic nature of the intertidal zone. Apparent sub-seasonal variability could also be a product of time-averaging introduced during sampling, especially for mussels living across a water depth gradient larger than 100 cm.

### Millennial variability

**Fig 9:**
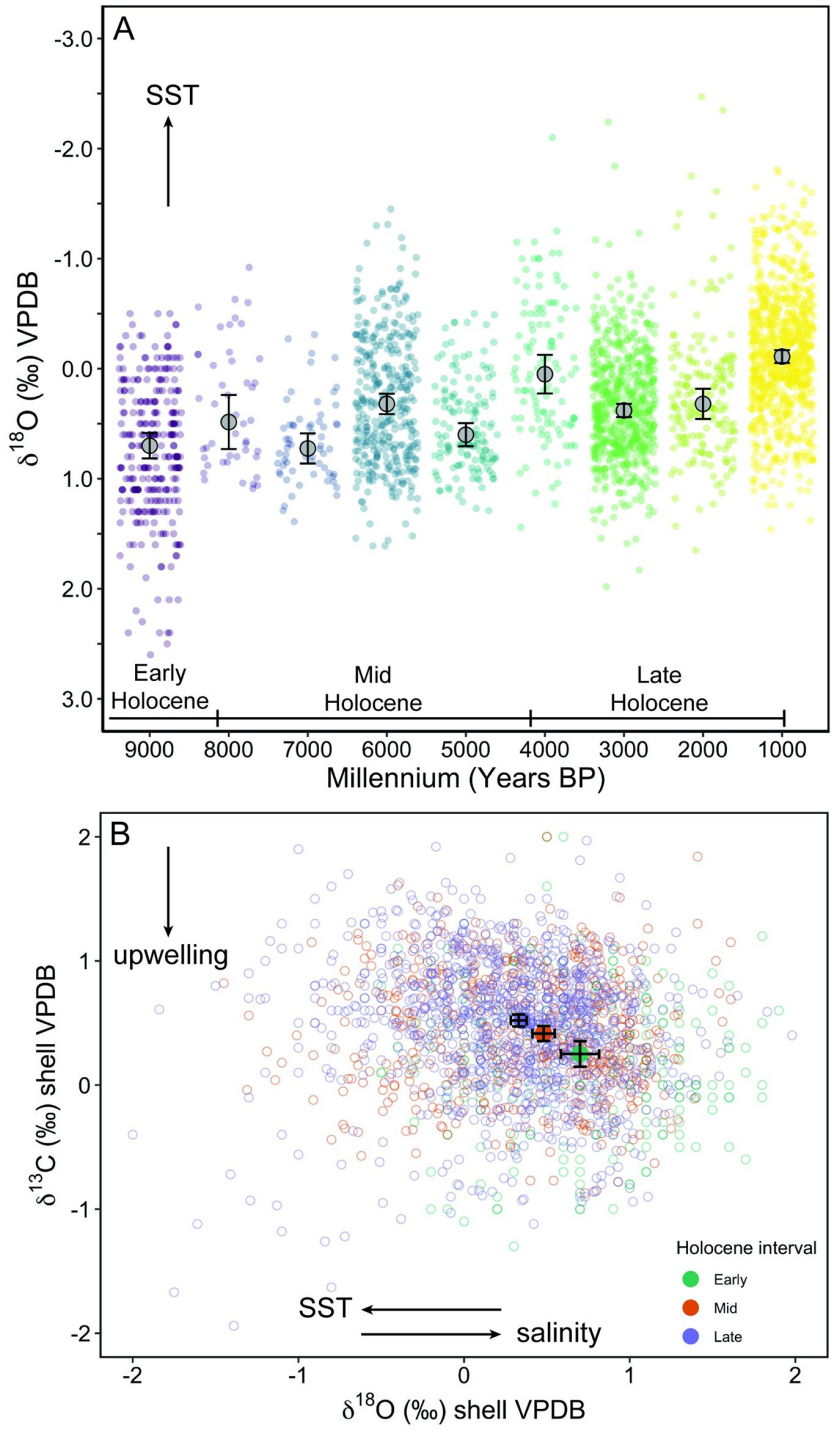
All data points from all Channel Islands shells with five or more subsamples binned by time interval. (A) Each time bin on the x-axis represents all samples within that millennium (e.g., 1000 represents all non-modern shells from 0–1000 BP, etc.). Gray points denote median δ^18^O_shell_ value of each time bin. Error bars represent three times the standard error (3SE) from the median for visibility. (B) All data points from Channel Islands shells in oxygen and carbon isotope space over the Holocene. Centroids (medians) are plotted for each sub-epoch. Both horizontal and vertical error bars represent 3SE from the median. Arrow shows expected directionality of a potential δ^13^C upwelling signal. Seven outliers with values greater than 2‰ or less than -2‰ were omitted from the plot.

Holocene climate in North America is characterized by millennial-scale temperature variability [109]. We aimed to determine whether *M*. *californianus* from the Channel Islands collectively recorded such millennial-scale variability despite the additional patterns that emerged in our record, such as local-scale oceanographic differences among the islands (Fig 5) or the ontogenetic and seasonal variability within individuals (Fig 7). To investigate millennial trends, we categorized the δ^18^O_shell_ data into 1000-year time bins using individuals with five or more subsamples (Fig 9), which revealed that median δ^18^O_shell_ values oscillate every 1000 years, with the highest median δ^18^O_shell_ values occurring during 9000–8000 BP and 7000–6000 BP (0.7‰ and 0.73‰, respectively) and the lowest median δ^18^O value (0.05‰) occurring at the start of the late Holocene (4000–3000 BP). ANOVA and Tukey HSD showed that millennial δ^18^O_shell_ values were statistically different from the previous millennium, except for from 8000 to 7000 BP and 3000 to 2000 BP. The statistical comparisons between each millennium are in the supporting information (S2 Table). Median δ^18^O_shell_ for modern (non-midden, live-collected) shells is 0.32‰, equal to median δ^18^O_shell_ values from 6000–5000 BP and 2000–1000 BP and lower than the median δ^18^O_shell_ value of six of the nine millennia since 9000 BP (Fig 9a). There has been an overall decline in collective median δ^18^O_shell_ values from the early Holocene to the late Holocene, indicating an overall warming and freshening signal over the three intervals (Fig 9b). Interestingly, the Channel Islands mussels collectively recorded millennial-scale shifts in addition to finer-scale trends (e.g., ontogeny, seasonality, local oceanography, micro-habitat), indicating that *M*. *californianus* shells provide a multi-scaled view of past environmental variability depending on the categorization of the data.

### Comparison to regional climate records

**Fig 10:**
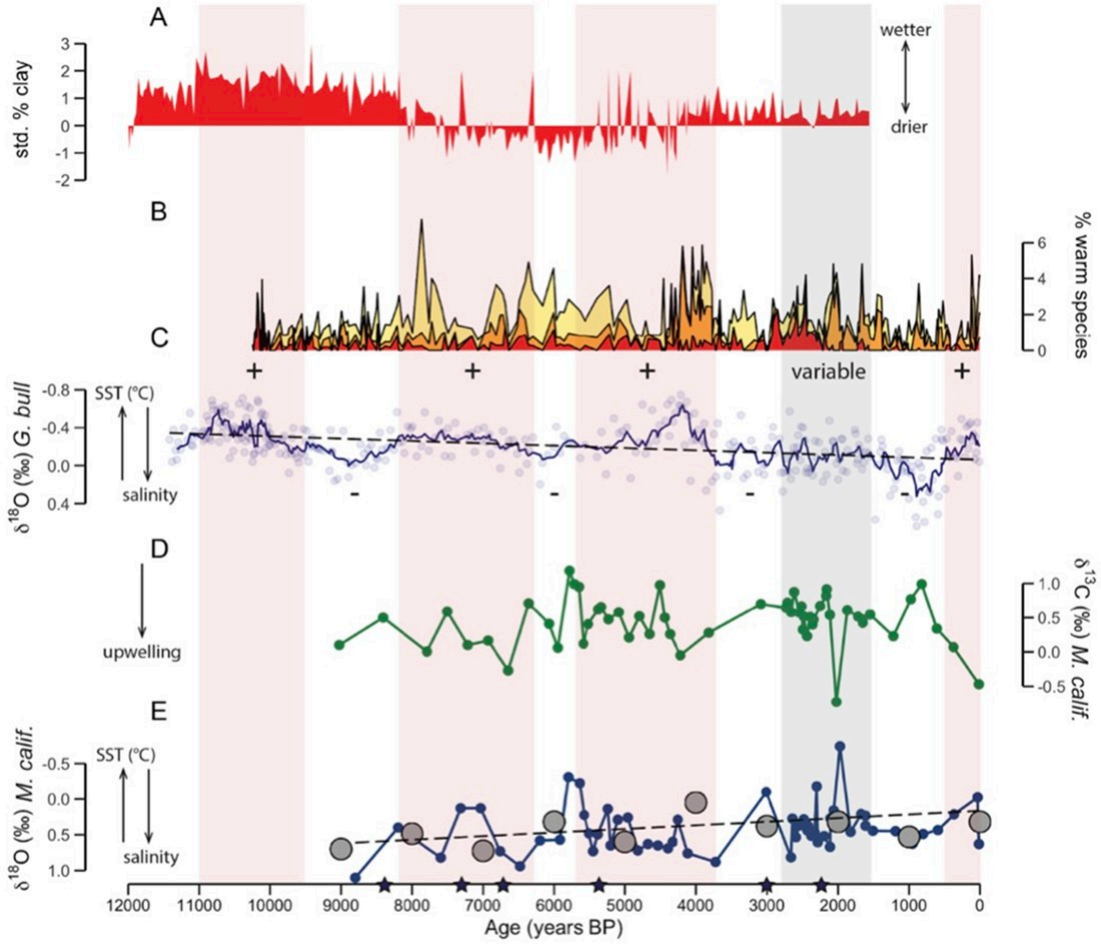
Age versus climate proxy data in calibrated years before present. (A) Standardized percent clay as a precipitation proxy from Silver Lake in the Central Mojave, California (Kirby et al., 2015) [110]. (B) Percentage of warm species (*Globigerinoides ruber* in orange, *Globoturborotalita rubescens* in red, and *Neogloboquadrina dutertrei* in yellow) from Santa Barbara Basin ODP Site 893 (Fisler and Hendy, 2008) [111]. (C) Ice-volume corrected oxygen isotope SST record from *Globigerina bulloides* planktic foraminifera at ODP Site 893 A/B (Kennett et al., 2007) [105]. Y-axis is inverted. Dashed line represents linear trend. (D) Carbon isotope record from *M*. *californianus* shells analyzed in this study with 5 or more subsamples with the median for each year of the record plotted. (E) Oxygen isotope record from *M*. *californianus* shells analyzed in this study with five or more subsamples with median for each year of the record plotted in blue. Y-axis is inverted. Dashed line represents linear trend. Large gray points represent 1000-year median δ^18^O_shell_ values as in Fig 9. Blue stars on x-axis denote timing of flood events identified from sediment cores in Santa Barbara Basin by Du et al. (2018) [112]. Shaded red bars denote warm periods and shaded gray bar denotes variable conditions inferred by Kennett et al. (2007) [105].

We generated a snapshot-based Holocene *M*. *californianus* stable isotope record and compared it to other published coeval climate records for the southern California region to determine whether this intertidal species is a comparable archive of broader climate patterns. We used all Channel Islands mussel shells with five or more subsamples (medium and long profiles) to calculate median δ^18^O_shell_ and δ^13^C_shell_ values for each year of the record to generate 58 annual snapshots over the Holocene (Fig 10). We calculated annual snapshots based on a San Diego study that found *M*. *californianus* shells to serve as reliable records of δ^18^O_shell_-inferred mean annual temperature in southern California [42]. We aimed to assess the reliability of the aggregated *M*. *californianus* shell isotope record when analyzed in this way. The annual snapshots were plotted along with 1000-year δ^18^O_shell_ medians (as in Fig 9a) as well as previously published terrestrial precipitation records [110] (Fig 10a) and marine sediment core records [105, 111] (Fig 10b and 10c) from southern California sites to determine whether *M*. *californianus* can be interpreted similarly for use as a comparable climate record for this region.

The annually resolved *M*. *californianus* oxygen isotope record (Fig 10e) does not closely match inferred temperatures from δ^18^O_foraminifera_ (Fig 10c) from varved sediments in the Santa Barbara Basin. There are three factors contributing to the misalignment between the *M*. *californianus* annual inferred SST record (Fig 10e) and the sediment core record (Fig 10c). Firstly, there are significant environmental differences (e.g., temperature, salinity, nutrients, etc.) between the offshore habitat of the planktic foraminifera *G*. *bulloides* and the intertidal habitat of *M*. *californianus*. Secondly, there are also major differences between the temporal resolutions of each record; varved marine sediments provide a much more temporally continuous record than *M*. *californianus* shells, while *M*. *californianus* presents a sparser record of longer-lived shells with nested seasonality and ontogenetic variability. Annual treatment of δ^18^O_shell_ data, such as calculating an annual average or annual median from multiple subsamples and individuals, may obscure the seasonal and ontogenetic patterns that we identified in individual shells. Sampling methods can also play a significant role in observed δ^18^O_shell_ patterns. Lastly, record misalignment is likely influenced by the chronological uncertainty resulting from differences in age model calibrations. While all archaeological shells were from a dated section of a midden, the source material and calibration curves varied. In some cases, the primary authors did not report the ΔR, or used a variety of ΔR values for the purposes of their study. The most commonly used ΔR value was 225 ± 35 years [52, 72, 105]. Annual binning of data as in Fig 10e is based on the author’s original interpretations for the purpose of demonstrating challenges and identifying possible sources of inconsistencies between the mussel record and the sediment core record.

Despite the challenges of interpreting an aggregated *M*. *californianus* geochemical dataset, it is still useful to examine the full *M*. *californianus* record at coarser resolution using large sample sizes, as supported by the statistically significant millennial-scale oscillations and geographic trends that emerge when *M*. *californianus* shells are analyzed collectively (Figs 5 and 9). Additionally, both the δ^18^O_shell_ and δ^18^O_foraminifera_ records underscore recent warming since ~ 1000 BP. In some cases, low annual δ^18^O_shell_ values, which can be indicative of low-salinity conditions, align remarkably well with Santa Barbara Basin flooding events [112], although the alignment is not consistent enough throughout the Holocene to indicate that mussels experience and reliably record freshwater input pulses as they calcify (Fig 10e). The lack of a discernible freshwater signal across the aggregated record corroborates the absence of any statistically significant positive δ^18^O_shell_-δ^13^C_shell_ correlation within individual shells (S4 Fig).

### Summary of confounding factors

Several environmental and biological factors influence shell chemical compositions, and therefore impact the fidelity of stable isotope data. A complete and reliable shell stable isotope record requires constant and consistent shell growth and high-resolution subsampling along the ontogenetic trajectory of the shell. Constant and consistent growth is not likely in *M*. *californianus* due to interruptions in its highly dynamic intertidal habitat and its own biological processes. Therefore, to optimize the paleoceanographic and paleobiological utility of the *M*. *californianus* stable isotope record, it is necessary to examine the confounding factors and develop methods to extract the signal or parameter of interest (Table 5). To reconstruct temperature seasonality from an individual shell, is it critical to determine that the controls on δ^18^O_shell_ are temperature-dependent and that the organism was growing throughout the annual cycle. In many cases, environmental parameters (e.g., temperature, seasonality, salinity) interact with biological factors (e.g., calcification, metabolism) to obscure or exaggerate signals. For example, growth cessation during cold conditions would obscure a winter signal, while rapid growth during warm conditions would result in an overrepresented summer signal. Based on our synthesis of stable isotope shell data from a variety of sites, ages, and sampling methods, we identified four recurring factors–calcification, metabolism, ontogeny, and habitat–and proposed corresponding methodological recommendations to investigate and characterize them more accurately (Table 5).

**Table 5:** Identified factors that may impact environmental interpretations from *M*. *californianus* stable isotope data.

Factor	Impacts	Methodological recommendations
Calcification	Calcification cessation or slowdown inhibits the recording ability of ambient environmental conditions and biases the stable isotope record.	Compare stable isotope profiles of closely spaced subsamples across many individuals to identify recurring life-history patterns. Search for cuspate cyclicity of δ^18^O_shell_ and determine whether the cusps occur during inferred-warm or inferred-cool seasons to identify temperature-controlled calcification patterns. Compare stable isotope profiles from many individuals to synchronous geochemical records from subtidal calcifying species, which are more likely to calcify more consistently, at the same locality.
Metabolism	Slowed metabolism during stressful events may reduce, stop, or speed up shell growth and therefore alter the organism’s ability to record environmental conditions for a snapshot of time.	Sample serially along the full ontogenetic profile (umbo to commissure) to get a complete profile of variability experienced throughout the individual’s life. Limit environmental interpretations based on fewer than five serial samples in the shell. Compare δ^18^O_shell_ and δ^13^C_shell_ profiles to examine covariation. Intra-individual profiles that do not covary or covary in unexpected ways (e.g., low δ^13^C_shell_ aligning with low δ^18^O_shell_) may indicate the incorporation of isotopically light metabolic carbon.
Ontogeny	Typical ontogenetic patterns result in a reduction in shell growth as the organism ages. Slower or sporadic shell growth inhibits the organism’s ability to record ambient environmental conditions.	Sample serially along the full ontogenetic profile (umbo to commissure) across multiple individuals of a variety of sizes to confirm that the ontogenetic signal is as expected (decrease in wavelength of δ^18^O_shell_ profile as the organism ages). Environmental interpretations made at the start or end of the organism’s life should be limited, as they may be heavily influenced by ontogenetic growth patterns.
Habitat	The range of intertidal habitats of *M*. *californianus* introduces significant variability to shell growth patterns, and therefore stable isotope compositions, within the same population. Organisms that spend less time submerged in water likely calcify less, and therefore record less environmental information.	Analyze modern conspecific shells from the same location across a known intertidal gradient to establish a baseline of stable isotope ranges for individuals from different sub-zones across the intertidal zone at a specific site. Avoid averaging multiple subsamples from one or many individuals to obtain a single value; the stable isotope profile is best used as an indicator of cyclicity experienced by the individual rather than as an absolute measurement.

Methodological recommendations are based on the synthesis of *M*. *californianus* data analyzed here and are not exhaustive.

Despite the limitations with environmental interpretations, the *M*. *californianus* stable isotope record did align with expected spatial oceanographic conditions and seasonal cycles when the sampling methodology allowed. Long δ^18^O_shell_ profiles provided sufficient temporal context to reveal sub-annual and seasonal trends over the course of a mussel’s life, but short profiles indicate that fine-resolution variability is difficult to extract from 2–4 subsamples from one margin of the shell. For example, mussel shell chemistry across many medium and long subsample profiles from modern shells did appear to reflect the geographic temperature gradient between mainland southern California and the northern Channel Islands. This δ^18^O_shell_ record implied cooler conditions from east to west within the Channel Islands, which matches known seawater temperature datasets and reflects the competing influences of the cold California Current and the warmer California Undercurrent and Davidson Current.

Additionally, we found that five sequential subsamples per individual shell revealed a local δ^18^O_shell_ maximum and a local δ^18^O_shell_ minimum from which a winter low temperature and a summer high temperature could be inferred, respectively. While a five-sample profile appeared to estimate one annual temperature cycle, we emphasize the value of collecting as many sequential subsamples along the growth trajectory of the shell as possible to account for ontogenetic variation and capture multi-season or multi-year δ^18^O_shell_ profiles. A longer δ^18^O_shell_ profile allows for the identification of annual minima and maxima over multiple years, and sampling multiple individuals allows for the cross-correlation and corroboration of seasonal cycles within the same region and could potentially aid in the identification of ontogenetic or metabolic influences on shell growth. Season of harvest information will also be more reliable when there is a multi-seasonal or multi-annual record experienced by the mussel to contextualize end-of-life signals. Conversely, short δ^18^O_shell_ and δ^13^C_shell_ profiles comprising only a few subsamples at the terminal edge of the shell are less useful for paleo-seasonal and paleoceanographic reconstruction because they reflect shorter periods of time (e.g., weeks to months) and therefore lack the temporal duration required to identify the organism’s life-history patterns and reconstruct broader climate conditions occurring over multiple seasons or years. End-of-life shell chemistry in an aging individual is not necessarily reflective of ambient conditions due to ontogenetic declines in calcification. We also observed high δ^18^O_shell_ variability among specimens growing at the same time and location, which reduces confidence in δ^18^O-inferred temperature calculations from shells with terminal-edge sampling only. Lastly, we found inconsistent and unexpected δ^18^O_shell_-δ^13^C_shell_ relationships, affirming that the influences of sampling strategy, growth rate, and conditions at a given site or time interval can severely impact shell chemistry, and subsequently, paleoenvironmental interpretations.

## Conclusions

We integrated and synthesized δ^18^O and δ^13^C data from modern and archaeological *M*. *californianus* shells and found that this intertidal species collectively records local-scale oceanographic conditions and serves as an archive of temperature seasonality when the stable isotope subsampling approach captures multiple seasons of growth across many individuals. The *M*. *californianus* stable isotope record is complex to interpret due to multiple environmental and biological influences on shell chemistry. These challenges are compounded by the dynamic nature of micro-environments across the intertidal zone and the nested temporal scales (e.g., seasons within years within millennia) that must be disentangled to accurately interpret the record. Consequently, *M*. *californianus* provides different information from existing terrestrial and offshore proxy-based climate records and cannot be interpreted in the same way. However, it is possible to infer seasonal and oceanographic patterns by performing high-resolution ontogenetic serial sub-sampling and analysis of both δ^18^O_shell_ and δ^13^C_shell_ profiles to disentangle life-history trends from environmental signals. When analyzed in this way, archaeological *M*. *californianus* shells supplement existing climate records by providing seasonally resolved snapshots that are ~ 2–5 years in length, depending on the sampling method, shell length, and ontogenetic age at the date of harvest. We encourage the use of our sampling recommendations and further analysis of our synthesized datasets to reveal both paleoceanographic information, such as pre-industrial ranges of chemical variability, ocean circulation patterns, and paleo-seasonality, and biological information, such as shell life-history traits through time. We also encourage the use of the vast collections of published stable isotopic data from archaeological and fossil shells as paleoceanographic and paleobiological archives [e.g., 113, 114], and we emphasize the value of compiling and utilizing data syntheses of shell material to streamline usage for multiple disciplines while reducing the need for further invasive sampling of culturally significant sites.

## Land and data acknowledgment

We acknowledge that this paper references Indigenous peoples of California who have stewarded the land and sea for millennia. We aim to document biogeochemical variability through the lens of a culturally significant mollusc species without further destruction of culturally sacred sites, but we acknowledge that the data used here from previously published studies may have been originally acquired without consent from tribes. We acknowledge the keepers of intergenerational Indigenous knowledge who maintain stories and data of the impacts of settler colonization on humans, ecosystems, and global climate. We direct readers to the open-source resource: https://native-land.ca/ as a starting point to identify the homelands of the diverse Indigenous peoples of this region. Finally, we encourage the use of existing datasets and the prioritization of noninvasive methods in accordance with intersectional values, perspectives, and ethics of multiple communities.

## Supporting information

S1 Fig Plots comparing subsampling strategies in individuals from the same ^14^C age. Out of all *M*. *californianus* shells synthesized here, there were only three cases where we could evaluate the impacts of subsampling strategy. We compared mussels collected from the same site and with the same ^14^C age (cal BP) with different subsampling strategies (long vs. medium profiles, each one in a different individual). Each plot is a different individual. Top panels show longer profiles and bottom panels show medium length profiles. Blue horizontal line represents the mean δ^18^O_shell_ value for that individual. The only case where the mean δ^18^O value is significantly different is for the two individuals from 5380 BP. Y-axes are inverted.
(PNG)

S2 Fig Oxygen (blue) and carbon (green) isotope profiles for long-profile *M*. *californianus* shells from the Channel Islands of all ages. Each plot is an individual shell. This figure is comparable to Fig 7, which has δ^18^O_shell_ profiles for the same individuals plotted. This figure shows δ^13^C profiles in addition.
(PNG)

S3 Fig Short δ^18^O_shell_ profiles from the 76 individuals live-collected at Santa Rosa Island in August 2017 (Jazwa et al., 2020) [74]. Out of the 76 individuals, 48 record overall summer warming throughout the profile. Both x- and y- axes are uniform for all individuals and the y-axis is inverted to match directionality of δ^18^O_shell_ inferred temperature (i.e., summer warming should appear as an increasing curve from left to right).
(PNG)

S4 Fig δ^18^O_shell_-δ^13^C_shell_ relationships among the 28 long-profile individuals. Linear regressions are plotted; a positive δ^18^O_shell_-δ^13^C_shell_ correlation is indicative of freshwater input and a negative δ^18^O_shell_-δ^13^C_shell_ correlation is indicative of upwelling. The age (years BP) of each individual is labeled at the top of each individual’s plot. This figure is comparable to Fig 7 and S2 Fig, which feature the same 28 individuals as plotted here.
(PNG)

S1 File Synthesized dataset of all stable isotope shell data compiled and analyzed in this study. (XLSX)

S1 Table Statistical test results (ANOVA, Tukey HSD) of oxygen isotope data binned by site. Table includes significant differences between sites across the full study period.
(PDF)

S2 Table Statistical test results (ANOVA, Tukey HSD) of oxygen isotope data binned by millennia. Table includes significant differences occurring over millennial scales.
(PDF)
