# Supplementary figures and images for: Interpreting life-history traits, seasonal cycles, and coastal climate from an intertidal mussel species: Insights from 9000 years of synthesized stable isotope data
Source: PLoS One. 2024 May 22;19(5):e0302945. doi: 10.1371/journal.pone.0302945 (PMC11111024; doi:10.1371/journal.pone.0302945)

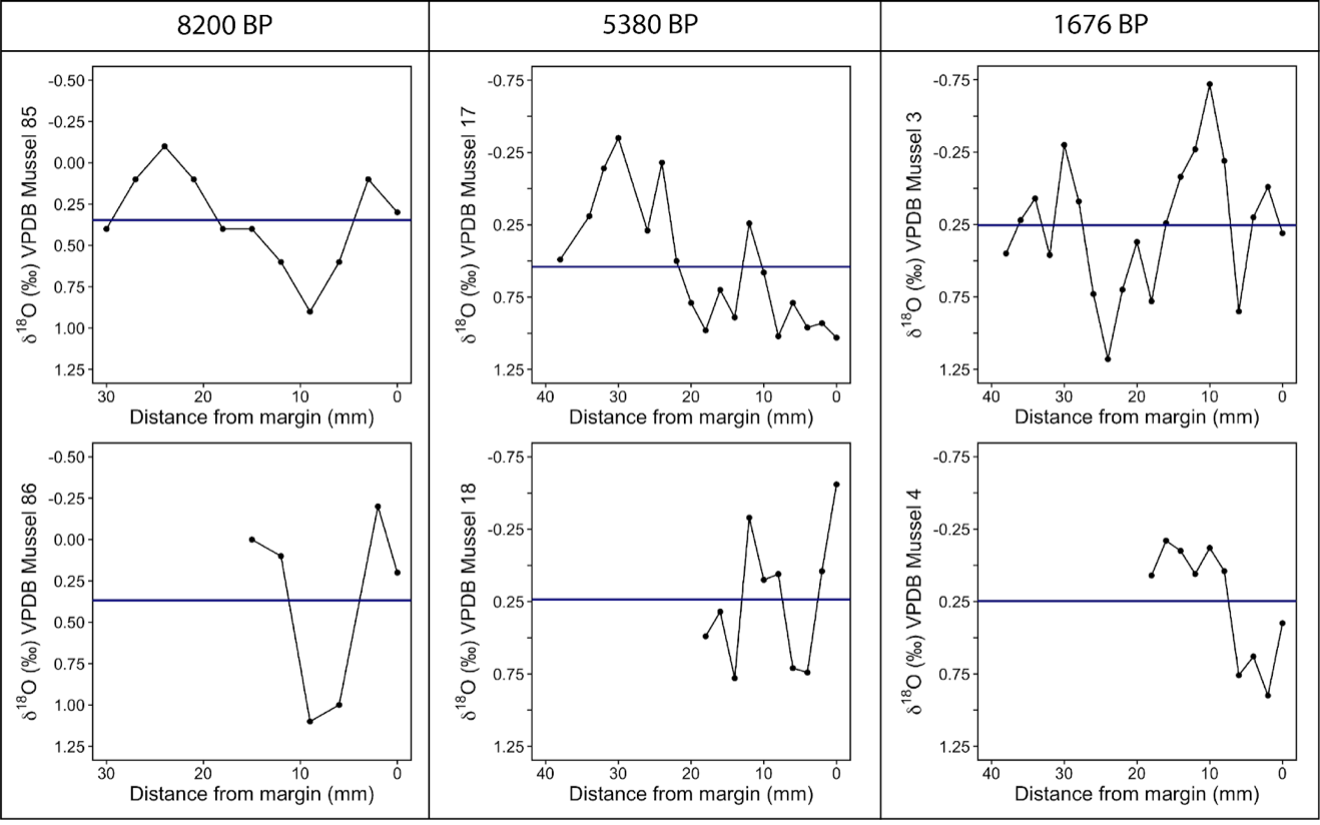

Supplement: S1 Fig — Out of all M. californianus shells synthesized here, there were only three cases where we could evaluate the impacts of subsampling strategy. We compared mussels collected from the same site and with the same 14C age (cal BP) with different subsampling strategies (long vs. medium profiles, each one in a different individual). Each plot is a different individual. Top panels show longer profiles and bottom panels show medium length profiles. Blue horizontal line represents the mean δ18Oshell value for that individual. The only case where the mean δ18O value is significantly different is for the two individuals from 5380 BP. Y-axes are inverted. (PNG) [file pone.0302945.s001.png]

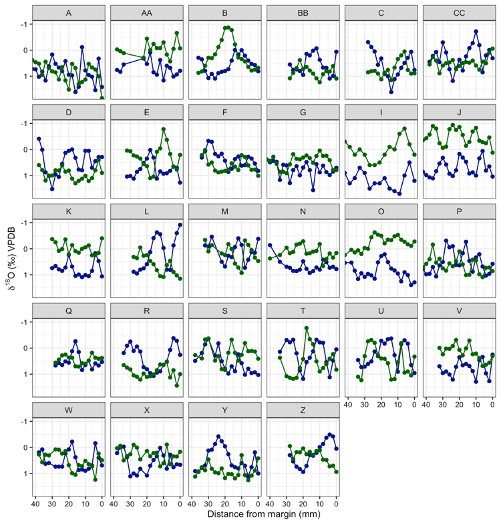

Supplement: S2 Fig — Each plot is an individual shell. This figure is comparable to Fig 7, which has δ18Oshell profiles for the same individuals plotted. This figure shows δ13C profiles in addition. (PNG) [file pone.0302945.s002.png]

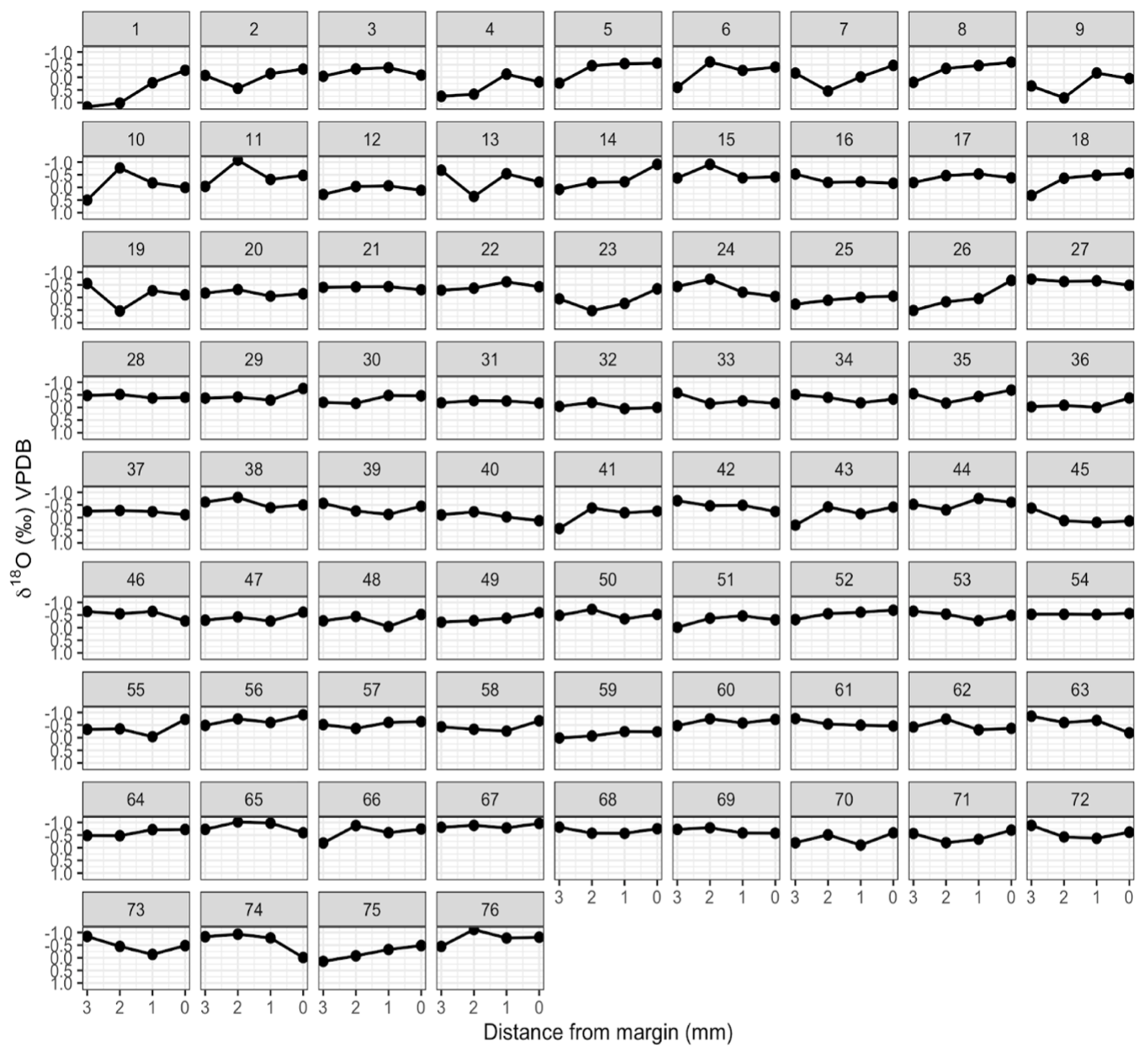

Supplement: S3 Fig — Out of the 76 individuals, 48 record overall summer warming throughout the profile. Both x- and y- axes are uniform for all individuals and the y-axis is inverted to match directionality of δ18Oshell inferred temperature (i.e., summer warming should appear as an increasing curve from left to right). (PNG) [file pone.0302945.s003.png]

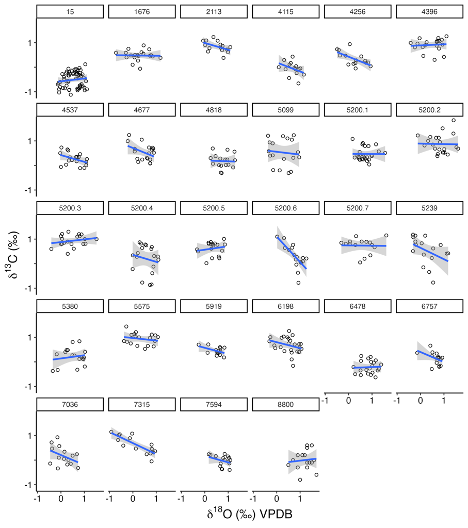

Supplement: S4 Fig — Linear regressions are plotted; a positive δ18Oshell-δ13Cshell correlation is indicative of freshwater input and a negative δ18Oshell-δ13Cshell correlation is indicative of upwelling. The age (years BP) of each individual is labeled at the top of each individual’s plot. This figure is comparable to Fig 7 and S2 Fig, which feature the same 28 individuals as plotted here. (PNG) [file pone.0302945.s004.png]
