# Supplementary material for: Interpreting life-history traits, seasonal cycles, and coastal climate from an intertidal mussel species: Insights from 9000 years of synthesized stable isotope data
Source: PLoS One. 2024 May 22;19(5):e0302945. doi: 10.1371/journal.pone.0302945 (PMC11111024; doi:10.1371/journal.pone.0302945)
Supplement: S1 Table — Table includes significant differences between sites across the full study period. (PDF) [file pone.0302945.s006.pdf]

Site Comparison	Difference	Lower	Upper	p-value
Newport Beach-Anacapa Island	-0.3857408	-0.55731249	-0.214169	0
San Diego-Anacapa Island	-0.1540066	-0.28192638	-0.0260869	0.007958
San Miguel Island-Anacapa Island	1.0303559	0.87855159	1.18216018	0
Santa Cruz Island-Anacapa Island	0.4101456	0.29188230	0.52840882	0
Santa Rosa Island-Anacapa Island	0.5737590	0.44918330	0.69833464	0
San Diego-Newport Beach	0.2317341	0.08372479	0.37974349	0.0001215
San Miguel Island-Newport Beach	1.4160966	1.24701723	1.58517607	0
Santa Cruz Island-Newport Beach	0.7958863	0.65613833	0.93563432	0
Santa Rosa Island-Newport Beach	0.9594997	0.81437082	1.10462865	0
San Miguel Island-San Diego	1.1843625	1.05980547	1.30891954	0
Santa Cruz Island-San Diego	0.5641522	0.48380227	0.6445021	0
Santa Rosa Island-San Diego	0.7277656	0.63838435	0.81714685	0
Santa Cruz Island-San Miguel Island	-0.6202103	-0.7348279	-0.5055928	0
Santa Rosa Island-San Miguel Island	-0.4565969	-0.57771705	-0.3354768	0
Santa Rosa Island-Santa Cruz Island	0.1636134	0.08870185	0.23852498	0
