# Supplementary material for: Interpreting life-history traits, seasonal cycles, and coastal climate from an intertidal mussel species: Insights from 9000 years of synthesized stable isotope data
Source: PLoS One. 2024 May 22;19(5):e0302945. doi: 10.1371/journal.pone.0302945 (PMC11111024; doi:10.1371/journal.pone.0302945)
Supplement: S2 Table — Table includes significant differences occurring over millennial scales. (PDF) [file pone.0302945.s007.pdf]

Millennium Comparison (Year BP)	Difference	Lower	Upper	p-value
1000-2000	-0.36938959	-0.51012044	-0.22865873	0
1000-3000	-0.48262339	-0.57421929	-0.39102749	0
1000-5000	-0.6622544	-0.81754606	-0.50696274	0
1000-6000	-0.43344712	-0.5420085	-0.32488574	0
1000-7000	-0.81624042	-1.03863233	-0.5938485	0
1000-8000	-0.50189259	-0.76152431	-0.24226086	0.0000001
1000-9000	-0.89773295	-1.01831315	-0.77715275	0
2000-4000	0.22941004	0.02508188	0.4337382	0.0146938
2000-5000	-0.29286481	-0.48457084	-0.10115879	0.0000777
2000-7000	-0.44685083	-0.69603723	-0.19766443	0.000001
2000-9000	-0.52834337	-0.69319265	-0.36349408	0
3000-4000	0.34264385	0.16847545	0.51681225	0
3000-5000	-0.17963101	-0.33880335	-0.02045866	0.0138082
3000-7000	-0.33361702	-0.55873587	-0.10849817	0.0001528
3000-9000	-0.41510956	-0.54064808	-0.28957104	0
4000-5000	-0.52227486	-0.73689154	-0.30765817	0
4000-6000	-0.29346757	-0.47712594	-0.10980921	0.0000264
4000-7000	-0.67626087	-0.94347424	-0.4090475	0
4000-8000	-0.36191304	-0.66083353	-0.06299256	0.0054686
4000-9000	-0.75775341	-0.94876232	-0.56674449	0
5000-6000	0.22880728	0.0593033	0.39831126	0.000961
5000-9000	-0.23547855	-0.41292041	-0.0580367	0.001296
6000-7000	-0.3827933	-0.61533203	-0.15025457	0.0000122
6000-9000	-0.46428583	-0.60268989	-0.32588177	0
8000-9000	-0.39584036	-0.66929739	-0.12238334	0.000251
7000-8000	0.31434783	-0.01685803	0.64555369	0.0785502
1000-4000	-0.13997955	-0.31060866	0.03064957	0.2102237
2000-3000	-0.11323381	-0.25823557	0.03176795	0.2710046
5000-7000	-0.15398601	-0.41167612	0.10370409	0.644971
5000-8000	0.16036181	-0.13007693	0.45080055	0.738025
2000-8000	-0.132503	-0.41542408	0.15041807	0.8764606
3000-6000	0.04917627	-0.06486717	0.16321972	0.9196979
2000-6000	-0.06405753	-0.22033061	0.09221554	0.9392879
7000-9000	-0.08149254	-0.31987935	0.15689428	0.9794916
6000-8000	-0.06844547	-0.33681971	0.19992877	0.9971079
3000-8000	-0.0192692	-0.28124051	0.24270212	0.9999998
